# Animal models for bipolar disorder: from bedside to the cage

**DOI:** 10.1186/s40345-017-0104-6

**Published:** 2017-10-13

**Authors:** Dominik K. E. Beyer, Nadja Freund

**Affiliations:** Experimental and Molecular Psychiatry, LWL University Hospital, Ruhr University Bochum, Universitätsstr. 150, 44801 Bochum, Germany

**Keywords:** Translational, Human condition, Circadian rhythm, Dopamine, Immune system, Stress

## Abstract

Bipolar disorder is characterized by recurrent manic and depressive episodes. Patients suffering from this disorder experience dramatic mood swings with a wide variety of typical behavioral facets, affecting overall activity, energy, sexual behavior, sense of self, self-esteem, circadian rhythm, cognition, and increased risk for suicide. Effective treatment options are limited and diagnosis can be complicated. To overcome these obstacles, a better understanding of the neurobiology underlying bipolar disorder is needed. Animal models can be useful tools in understanding brain mechanisms associated with certain behavior. The following review discusses several pathological aspects of humans suffering from bipolar disorder and compares these findings with insights obtained from several animal models mimicking diverse facets of its symptomatology. Various sections of the review concentrate on specific topics that are relevant in human patients, namely circadian rhythms, neurotransmitters, focusing on the dopaminergic system, stressful environment, and the immune system. We then explain how these areas have been manipulated to create animal models for the disorder. Even though several approaches have been conducted, there is still a lack of adequate animal models for bipolar disorder. Specifically, most animal models mimic only mania or depression and only a few include the cyclical nature of the human condition. Future studies could therefore focus on modeling both episodes in the same animal model to also have the possibility to investigate the switch from mania-like behavior to depressive-like behavior and vice versa. The use of viral tools and a focus on circadian rhythms and the immune system might make the creation of such animal models possible.

## Background

Bipolar disorder (BD) is characterized by recurrent episodes of manic and depressive states with intervening episodes of euthymia (normal mood) (Merikangas et al. 2007; Anderson et al. 2012; Phillips and Kupfer 2013). The symptomatology of BD is very heterogenic and heavily depends on the patient’s state. Throughout manic episodes, people undergo euphoria, aggression, reduced need for sleep, high reward seeking, hypersexuality, and hyperactivity (Perry et al. 2010; Anderson et al. 2012; Cheniaux et al. 2014). In contrast, a state of depression includes anhedonia, increased sleep, reduced libido, feeling tired, and a greater risk of suicide among other symptoms (American Psychiatric Association 2013; Anderson et al. 2012). In addition, BD patients suffer from various cognitive deficits (Martínez-Arán et al. 2004; Savitz et al. 2005; Burdick et al. 2007; Goodwin et al. 2008). Established treatments for BD include mood stabilizers, such as lithium, anticonvulsants, like valproate, and antipsychotics. Current treatments, however, are not able to completely stabilize behavioral aberrations or to recover cognitive deficits (Grunze et al. 2013; van Enkhuizen et al. 2015a). Lithium, the first line medication for BD, is furthermore suspected to cause negative side effects, including cognitive impairment (Pachet and Wisniewski 2003; Holmes et al. 2008; Grunze et al. 2013). Besides the lack of novel developed therapeutics (Malkesman et al. 2009), current clinical criteria fail to diagnose milder symptoms of BD and therefore it can take years to finally diagnose this psychiatric disorder (Merikangas et al. 2011). Improvement of treatment and diagnosis options is crucial, particular given the high rate of suicide attempts in patients with BD (Novick et al. 2010). A better understanding of cause and pathophysiology of the disease is needed to archive these improvements. Adequate animal models for BD will provide a useful tool to advance the knowledge on the underlying neurobiology of BD. Establishing animal models for psychiatric disorders, however, is a difficult task (Malkesman et al. 2009). Not only are the symptoms in patients with one disorder often quite broad and variable between patients, some symptoms used to diagnose psychiatric disorders in humans are not even possible to asses in animals, such as feelings of worthlessness or guilt (Malkoff-Schwartz et al. 1998; Nestler and Hyman 2010). Given its cyclical nature, BD is thereby especially hard to model (Gould and Einat 2007, 2014). So far BD is mainly investigated in separated animal models for either mania or depression (Einat 2014; van Enkhuizen et al. 2015a). The animal models mostly mimic some behavioral characteristics of BD, which are more or less easy to measure, such as overall locomotor activity, sexual behavior, aggression, risk taking, and decision making (Einat 2014; van Enkhuizen et al. 2015a; Harrison et al. 2016; Sharma et al. 2016). An animal model of BD in rodents, which models the whole complexity of symptoms, might never be possible. Nonetheless, it is still crucial to create and characterize new models for BD, which consistently tighten the gap between human pathophysiology and BD-like symptoms in animals. Animal models are an indispensable tool and a critical component in the preclinical research field. They allow developing new treatment and diagnosis options and therewith improving the lives of BD patients and can therefore not be replaced. Ideally, an adequate animal model of BD should include elements of the three axes of validity: face, predictive, and construct validity. Face validity indicates to which extent the model reflects characteristics of the human disease. Predictive validity refers to which degree the model will respond to an efficient treatment in humans. Construct validity reflects to which extent the model measures what it claims to be measuring (Einat 2014; Malkesman et al. 2009). The ideal animal model should therefore comply with the following requirements: (i) model BD-specific behavioral abnormalities with ideally all its facets; (ii) consider the cyclical nature of BD; (iii) be able to spontaneously switch between both episodes (all face validity); (iv) respond to current established treatment; and (v) due to the fact that not all BD patients respond to medications (Cipriani et al. 2011), reflect a distribution of responders and nonresponders (both predictive validity) (Einat 2014). This division would represent the result of clinical trials and therefore a group effect of treatment should still be observed. Additional prior determination of individual, untreated baseline measurements will be required to compare intra-individual differences before and after treatment of the animals to later successfully separate responders from nonresponders. It should furthermore (vi) be affected by pharmacological, environmental, or genetically manipulations in regard to the same mechanisms that are involved in human patients (face validity).

To date, no such animal model for BD exists. However, within the last couple of years several models were able to at least address some of the above-mentioned requirements and have therewith been able to improve our understanding of the neurobiology underlying BD. In this review, we will discuss various aspects that are affected in patients with BD, namely circadian rhythm, neurotransmitters focusing on the dopaminergic system, environment, and immune system. Disruptions and influences regarding these topics in patients will be compared to findings in animal models and we will illustrate how these findings have been used to develop animal models for the disorder.

### Circadian rhythm

Aberrations of the sleep–wake cycle and circadian rhythms belong to primary symptoms of patients suffering from BD and are used as diagnostic criteria (Gonzalez 2014; Kripke et al. 2009; McCarthy and Welsh 2012; Wirz-Justice 2006). Patients display irregularities in daily biological rhythms including sleep, activity, body temperature, hormonal secretions, cell regeneration, and eating behavior (Bunney and Potkin 2008; Goetze and Tölle 1987; McClung 2007; Salvatore and Tohen 2007; Souetre et al. 1988; Takahashi et al. 2008). In addition, psychotherapeutic treatment (interpersonal and social rhythm therapy) with the aim of stabilizing and structuring daily routines and thereby enabling a normalized sleep–wake cycle is an effective therapeutic tool for mood stabilization and can reduce the number of manic and depressive episodes (Frank et al. 2000, 2007; Miklowitz et al. 2007). At the same time, mania can be induced by disruption of circadian rhythms (Bunney and Bunney 2000; McClung 2013). Circadian disruptions present in humans suffering from BD suggest an involvement of circadian clock genes in the pathogenesis of the disease (Cosgrove et al. 2016; Etain et al. 2011; Wirz-Justice 2006; McClung 2007; Frank et al. 2000). Indeed, BD symptoms are correlated with disruptions of the circadian rhythm and associated with a polymorphism of the *circadian locomotor output cycles kaput* (*Clock*) gene (Benedetti et al. 2003; Logan and McClung 2016; Serretti et al. 2003).

Targeting circadian rhythm genes to disrupt mechanisms regulating the circadian rhythm has been widely used to create animal models for BD (McClung et al. 2005; Mukherjee et al. 2010; Roybal et al. 2007). The master pacemaker of the circadian rhythm is localized in the suprachiasmatic nuclei and interconnects a complex network of transcriptional–translational activation and repression, resulting in an oscillating expression of clock genes over a period of 24 h (Takahashi et al. 2008). Several diverse preparations of *Clock* manipulation were used as animal models to study BD. The most common model is the *ClockΔ19* mutant mouse. These mice carry a deletion at exon 19 of the *Clock* gene, resulting in a dominant-negative protein, unable to activate transcription (King et al. 1997). Mutant mice exhibit mania-like behavior (Roybal et al. 2007) and altered sleep patterns (McClung 2013). The disruption of CLOCK resulted in lower immobility in the forced swim test, a greater preference for rewarding stimuli, such as sucrose solution and cocaine, a lower threshold within intra-cranial self-stimulation at lower drug doses, lowered anxiety levels, and less depressive-like behavior (McClung et al. 2005; Roybal et al. 2007). In addition, the *Clock* mutant mice exhibited deficits within the paired pulse inhibition paradigm (van Enkhuizen et al. 2013b). *ClockΔ19* mutant mice were also tested in the behavioral pattern monitor (BPM), a test to pattern and level of locomotor activity, exploratory behavior, and novelty seeking in humans and rodents (Perry et al. 2009; Young et al. 2007). While BD patients show increased exploration and goal-directed behavior, illustrated through linear and direct movements (Logan and McClung 2016; Minassian et al. 2011; Perry et al. 2009, 2010), the *ClockΔ19* mutant mice do not represent this specific exploration and goal-directed behavior. They exhibit more circumscribed, small-scale movements (Perry et al. 2009; van Enkhuizen et al. 2013b). In summary, the *ClockΔ19* mutant mice resemble various but not all behavioral aspects of BD mania in humans to its full extent. Another manipulation of *Clock*, which resulted in BD-relevant behavior, is the knock-down of CLOCK specifically in the ventral tegmental area (VTA) of mice (Mukherjee et al. 2010). The knock-down of *Clock* expression resulted in abnormal circadian rhythms, indicated by less robust activity in dark phases and enhanced activity in resting phases, less anxiety behavior, and increased locomotor activity in a novel environment. Despite the observed hyperactivity in a novel environment, the overall locomotor activity over a period of 24 h, however, was reduced (Mukherjee et al. 2010). In contrast to the previously observed less-depression-like behavior of the *ClockΔ19* mutant mice (Roybal et al. 2007), the CLOCK knock-down mice exhibited increased depression-like behavior in the forced swim and learned helplessness test and thereby express a mixed state of mania- and depression-like behavior (Mukherjee et al. 2010). Mukherjee and colleagues postulated, therefore, that CLOCK’s functioning in the VTA is required for the regulation of mood-related behavior. This hypothesis is supported by the fact that over-expression of CLOCK in the VTA reduces hyperactivity and restores anxiety-related behavior almost to wild-type level in the *ClockΔ19* mutant mice (Roybal et al. 2007). Disrupted circadian rhythm in these animal models might create a vulnerable state with a greater sensitivity for addiction and mood disorders (Logan et al. 2014; Logan and McClung 2016). Commonly found in both *Clock* mice models is an enhanced dopamine (DA) release from neurons in the VTA, which is reflected, for example, in an increased dopaminergic cell firing rate (Coque et al. 2011; McClung et al. 2005; Mukherjee et al. 2010; Roybal et al. 2007). This functional linkage between the dopaminergic system (see “Dopaminergic pathways”) and aberrant circadian rhythms connects two major pathways, which may be involved in the pathogenesis of BD and are often used as targets for the development of genetic or environmental animal models for BD. This involvement of the dopaminergic system supports the dopamine hypothesis (Berk et al. 2007) that hyper-dopaminergic transmission might be responsible for mania in humans and therefore also for mania-like behavior in animals.

Chronic lithium treatment was able to normalize various aspects of aberrant behavior in the *ClockΔ19* mutant mice (Coque et al. 2011; Roybal et al. 2007). Lithium’s therapeutic efficacy might be due to its properties to lengthen the circadian period, which was observed across several species (Klemfuss 1992; Kripke et al. 1978). One well-studied potential target of lithium’s action is the inhibition of glycogen synthase kinase-3 beta (GSK-3β) (Klein and Melton 1996; Serretti et al. 2009; but see also Agam and Azab 2016). GSK-3β is involved in various cell functions, like gene transcription, neurogenesis, and apoptosis (Doble and Woodgett 2003). GSK-3β is also able to regulate the circadian clock through phosphorylation of CLOCK and nuclear receptor subfamily1, group D, member1 (REV-ERBα) (Bellet and Sassone-Corsi 2010; Besing et al. 2015; Martinek et al. 2001; Yin et al. 2006) and thereby modulates the circadian rhythm (Besing et al. 2015). Synthetic inhibition of GSK-3β was able to mimic the effects of lithium and to prevent mania-like behavior, such as amphetamine-induced hyperactivity, in male C57BL/6J mice (Kozikowski et al. 2007). In addition, *gsk*-*3β* haploinsufficient mutant mice, lacking one copy of the gene coding for GSK-3β, show the same behavioral effects as lithium-treated mice (O’Brien et al. 2004). *gsk*-*3β* haploinsufficiency reduces exploratory behavior and immobility time in the forced swim test, comparable to treatment with lithium in wild-type mice, without affecting overall activity (O’Brien et al. 2004). Both manipulations of GSK-3β suggest that lithium’s therapeutic effect as a mood stabilizer depends on inhibiting GSK-3β activity (O’Brien et al. 2011). Once again a manipulation of the circadian rhythm through transgenic mice over-expressing GSK-3β resulted in mania-like behavior (Prickaerts et al. 2006). The GSK-3β over-expressing mice exhibited hyperactivity, reduced immobility in the forced swim test, reduced habituation in the open field test, and increased acoustic startle response (Prickaerts et al. 2006). Patients in manic episodes opposingly exhibit reduced startle responses (Perry et al. 2001). Due to the nonspecific alterations of the dopaminergic system in the GSK-3β over-expressing mice, which are also recognizable in other psychiatric disorders, such as schizophrenia and attention-deficit hyperactivity disorder (ADHD), this animal model, however, lacks specificity for mania (Sharma et al. 2016).

An additional target of the circadian rhythm, which can be used to model BD-relevant behavior, is the extracellular-signal-regulated kinase (ERK) (Engel et al. 2008). The ERK pathway mediates proliferation, differentiation, and plasticity of neurons in the central nervous system (Thomas and Huganir 2004). ERKs are also involved in resetting the master pacemaker in the suprachiasmatic nucleus via photic input (Butcher et al. 2002; Coogan and Piggins 2004). Infusion of ERK inhibitor into the suprachiasmatic nucleus of mice prevents the activity rhythms shift, which is usually observed between light and dark phases (Butcher et al. 2002). A knock-out of the gene coding for ERK1 resulted in hyperactivity, enhanced goal-directed activity, increased risk taking or impulsivity, and increased reward-motivated behavior (Engel et al. 2008). In addition, the ERK1 pathway can be activated by lithium and valproate, but only valproate, not lithium, was able to reduce the behavioral abnormalities (Engel et al. 2008). The ERK pathway can in turn be activated by neurotrophins. One of these neurotrophins might play a role in the pathophysiology of BD, namely the brain-derived neurotrophic factor (BDNF) (Frey et al. 2013; Södersten et al. 2014). *BDNF* haploinsufficient mice indeed exhibit mania-like behavior, including hyperactivity, increased aggressive behavior, and appetite (Kernie et al. 2000; Lyons et al. 1999). Interestingly, even untreated *BDNF* haploinsufficient mice show reduced hippocampal volume and their CA3 dendritic arborizations resembled stressed wild-type mice, suggesting a role of BDNF in hippocampal dendritic remodeling (Magariños et al. 2011).

One downstream target of the ERK signaling pathway is B-cell lymphoma 2 (Bcl-2), which is involved in neuronal development, plasticity, and degeneration (Akhtar et al. 2004) through inhibition of apoptosis (Bold et al. 1999; Campani et al. 2001). Interestingly, lithium affects the Bcl-2 levels, with chronic lithium treatment increasing Bcl-2 levels in the brain of rats (Chen et al. 1999; Manji et al. 2000). Consistent with this effect of lithium is that transgenic over-expression of Bcl-2 in mice prevents neuronal death (Bonfanti et al. 1996) and acts protective against deleterious stress-induced neuronal endangerment (DeVries et al. 2001). Although BD is rather associated with neuroplasticity deficits than neurodegenerative events (Rajkowska 2002), cell death might play a role in the pathogenesis of BD (Lee et al. 2002). Bcl-2 manipulation might also be related to anxiety as mice with an additional *Bcl*-*2* transgene, and therefore elevated Bcl-2 levels, exhibited less anxiety behavior (Rondi-Reig et al. 1997; Rondi-Reig and Mariani 2002). On the other hand, mice with a heterozygous knock-out of the *Bcl*-*2* gene exhibit Bcl-2 deficiency and increased anxiety behavior (Einat et al. 2005). In addition, Bcl-2 heterozygous knock-out mice show some behaviors similar to mania, including increased reward seeking and amphetamine sensitization, and lithium pretreatment attenuated sensitization in these animals (Lien et al. 2008).

Another gene heavily involved in the regulation of circadian rhythms is *Dbp*. It encodes for the albumin D element-binding protein, a transcription factor that is regulated by the CLOCK protein (Ripperger et al. 2000; Wuarin et al. 1992). *Dbp* expression is affected in patients with BD and can furthermore be influenced by lithium treatment (Kittel-Schneider et al. 2015). A heterozygotous knock-out of DBP in mice induces a depressive-like phenotype indicated by reduced locomotor activity and diminished response to amphetamine. When exposed to environmental stress (see “Environment: stressors”), DBP knock-out mice show a switch in behavior and become hyperactive. This switch, which to some extend resembles the switch from depression to mania in BD patients, can be prevented by the administration of valproate (Le-Niculescu et al. 2008).

But even stressors alone (e.g., sleep deprivation) can disrupt the circadian clock resulting in changes of mood and even the induction of mania in BD patients (Colombo et al. 1999; Malkoff-Schwartz et al. 1998; Wright 1993). It is therefore possible that sleep deprivation paradigms can induce mania-like behavior in rodents. Indeed, wild-type rats after typically 72 h of sleep deprivation exhibited mania-like behavior, such as enhanced aggressive behavior and hypersexuality (Gessa et al. 1995; Hicks et al. 1979; Morden et al. 1968). But this behavioral phenotype lasted only for about 30 min. In addition, chronic lithium can reverse the mania-like behavior (Gessa et al. 1995). However, it should be noted that the disruption of regular sleep requires the usage of techniques that cause additional stress (i.e., immobilization, isolation, and the fear and experience of falling into water) (Logan and McClung 2016). Benedetti and colleagues used an improved protocol to minimize these additional stressors and still found mania-like behavior, such as increased locomotor activity and aggressive behavior (Benedetti et al. 2008). These results indicate that sleep deprivation alone is a sufficient stressor to induce BD-relevant behavior.

An additional possibility to affect the sleep–wake cycle is the high-frequency stimulation of the lateral hypothalamus, which resulted in mania-like behavior in rats (Abulseoud et al. 2014). The hypothalamic stimulated rats exhibited hyperactivity, such as increased grooming, and reduced resting phases, as well as hypersexuality, i.e., increased rearing and sexual self-stimulation. These behavioral characteristics could be attenuated through chronic lithium treatment.

Apart from the cycle of day and night, the changing of seasons and the associated photoperiod length can trigger changes in mood (Young and Dulcis 2015). Indeed, a seasonal pattern of the episodes of BD was identified in a proportion of patients (Schaffer et al. 2003), whereas depressive symptoms are more prevalent during winter months (Meesters and Gordijn 2016; Rosenthal et al. 1984). This seasonal effect might be due to shortening or lengthening of the day-lengths and the associated received illumination. Modified illumination in rats induced a switch in neurotransmitter expression. A long day period of 19 h of light resulted in a switch from DA to somatostatin expression in hypothalamic neurons after 1 week. The contrary effect was observed with a short day period of 5 h light (Dulcis et al. 2013). In addition, a matching pattern of receptor expression was observed: an increased expression of postsynaptic dopamine D2 receptors (D2R) was accompanied by the presynaptic increase in dopaminergic interneurons (Dulcis et al. 2013). Rats in the long day period exhibited more anxiety behavior in the elevated plus maze and more depressive-like behavior measured by increased immobile time in the forced swim test, whereas animals in the short day period displayed decreased anxiety-related behavior and less immobile time (Dulcis et al. 2013).

Disruption of the circadian rhythms has been widely used to induce BD-like behavior, mainly mania-like behavior (Table 1). Here it is to consider that the manipulations have been mainly conducted in nocturnal animals and results might not be comparable to mechanisms in diurnal humans (Challet 2007). Using diurnal rodent animal models could be beneficial in this field of research (Ashkenazy et al. 2009; Bilu et al. 2016; Einat et al. 2006; Leach et al. 2013). Nevertheless, effects are quite robust as various disruptions of one gene (i.e., *Clock*) as well as targeting related pathways or environmentally influence sleeping behavior result in similar effects (McClung et al. 2005; Mukherjee et al. 2010; Prickaerts et al. 2006; Roybal et al. 2007). Observations of depressive-like behavior after manipulations of circadian rhythms are rare. We see mixed behavior, some aspects of mania- and at the same time depressive-like behavior, in the *ClockΔ19* mutant mouse (Mukherjee et al. 2010). Similarly, manipulation of *Bcl*-*2*, a gene that has indirect connections with circadian rhythm pathways, can induce both behavioral states (Lien et al. 2008). Here it is important to note that elevated levels of Bcl-2 are associated with decreased anxiety (Rondi-Reig et al. 1997; Rondi-Reig and Mariani 2002), while Bcl-2 deficiency increases anxiety (Einat et al. 2005). The most promising model in terms of the cyclic characteristic of BD is the manipulation of length of day. Here we see depressive-like behavior when extending the day period and (at least) less depressive-like behavior when decreasing the day period (Dulcis et al. 2013).

**Table 1:** Summary of manipulable risk factors for BD and their influence on BD-relevant behavior

Manipulation	BD-relevant behavior	Neurobiology	References
*Circadian rhythm*			
*ClockΔ19* mutant mice	Hyperactivity	Altered sleep pattern	(Coque et al. 2011; van Enkhuizen et al. 2013b; McClung 2013; McClung et al. 2005; Mukherjee et al. 2010; Roybal et al. 2007)
	Altered sleep pattern	Enhanced DA release	
	Greater preference for rewarding stimuli		
	Decreased anxiety behavior		
	Less depressive-like behavior		
	Impaired PPI		
CLOCK knock-down mice	Abnormal circadian rhythms		
	Less anxiety		
	Hyperactivity in novel environment but decreased overall hyperactivity		
	Increased depression-like behavior and helplessness		
GSK-3β haploinsufficient mutant mice	Reduced exploration	Affect gene transcription, neurogenesis, and apoptosis	(Besing et al. 2015; O’Brien et al. 2004, 2011; Prickaerts et al. 2006)
	Less helplessness		
	Normal overall activity		
GSK-3β over-expression mice	Hyperactivity	Alterations of dopaminergic system	
	Less helplessness		
	Reduced habituation		
	Increased acoustic startle response		
ERK1 knock-out mice	Hyperactivity	Shift of activity rhythm	(Engel et al. 2008)
	Enhanced goal-directed activity		
	Increased risk taking and impulsivity		
	Increased reward seeking		
*BDNF* haploinsufficient mutant mice	Hyperactivity	Decreased BDNF level following DA overactivity	(Kernie et al. 2000; Lyons et al. 1999; Magariños et al. 2011)
	Increased aggression		
	Elevated appetite	Decreased hippocampal volume	
		CA3 dendritic arborizations resemble stressed wild-type mice	
Bcl-2 heterozygous knock-out mice	Increased anxiety	Decreased *Bcl*-*2* level	(DeVries et al. 2001; Einat et al. 2005; Lien et al. 2008; Rondi-Reig et al. 1997; Rondi-Reig and Mariani 2002)
	Increased reward seeking		
		Acts protective against deleterious stress-induced neuronal endangerment	
	Increased amphetamine sensitization		
DBP heterozygotous knock-out mice	Hypoactivity		(Le-Niculescu et al. 2008)
	Diminished response to amphetamine		
	Environmental stress induce hyperactivity		
Sleep deprivation	Hyperactivity		(Benedetti et al. 2008; Gessa et al. 1995; Hicks et al. 1979; Malkoff-Schwartz et al. 1998; Morden et al. 1968)
	Increased aggression		
	Increased exploratory behavior		
	Hypersexuality		
High-frequency stimulation of the lateral hypothalamus	Hyperactivity	Affects sleep–wake cycle	(Abulseoud et al. 2015; Abulseoud et al. 2014)
	Increased grooming		
	Hypersexuality		
	Reduced resting phases		
Photoperiod lengths	Anxiety behavior	Neurotransmitter switching	(Dulcis et al. 2013)
	Helplessness		
		(DA ↔ somatostatin)	
*Sensitization models*			
Administration of psychostimulants (amphetamine, cocaine)	Hyperactivity	Increased synaptic DA and NE levels	(Borison et al. 1978; Davies et al. 1974; Frey et al. 2006; Fries et al. 2015; Gould et al. 2001; Kilbey and Ellinwood 1977; Macêdo et al. 2012, 2013; Post 1992; Post 1990; Queiroz et al. 2015; Rezin et al. 2014; Rygula et al. 2015; Seiden et al. 1993; Zheng et al. 2013)
	Increased aggression		
		Disturbance of homeostatic mechanisms	
	Stereotypies		
	Increased hedonic behavior		
		Alterations in BDNF level	
	Disturbed sleep–wake cycle		
	Declined cognitive performance		
	Deficient PPI response		
Withdrawal following chronically psychostimulant administration	Hypoactivation	Supersensitivity of serotoninergic neurons a decrease in NE	(Barr et al. 1999; Barr and Phillips 1999, 2002; Baumann and Rothman 1998; Markou and Koob 1991; Marszalek-Grabska et al. 2016; Mutschler and Miczek 1998; Paulson et al. 1991; Schindler et al. 1994; Schwartz et al. 1982; Wise and Munn 1995)
	Increased anxiety		
	Anhedonia		
	Increased negative contrast		
		Reduced DA responsiveness	
	Decreased motivation		
*Dopaminergic pathways*			
Increased D1R expression in the prefrontal cortex	Increased impulsivity	Decreased D2R in nucleus accumbens	(Freund et al. 2016; Sonntag et al. 2014)
	Increased sexual behavior		
	Hedonic behavior		
	Addictive behavior		
Termination of previous D1R over-expression	Hypoactivity	Increased CREB in nucleus accumbens	(Freund et al. 2016)
	Anhedonic behavior		
	Helplessness		
DAT knock-down mice	Hyperactivity in novel environments	Hyperdopaminergia	(Dulcis et al. 2013; van Enkhuizen et al. 2014b; van Enkhuizen et al. 2014a; Giros et al. 1996; Ralph et al. 2001; Ralph-Williams et al. 2003; Young et al. 2010, 2011; Zhuang et al. 2001)
	Increased risk behavior		
	Hyperexploratory behavior		
	Less anxiety		
	Impaired decision making with a preference for high reward combined with high risk		
DAT knock-out mice	Hyperactivity		
	Sensorimotor deficits within PPI		
GluR6 knock-out mice	Hyperactivity		(Shaltiel et al. 2008)
	Increased risk taking		
	Elevated aggression		
	Heightened responsivity to amphetamine		
	Less anxiety		
*Environmental stressors*			
Prenatal stress	Hyperactivity in novel environment	Incomplete development of hippocampus and reduced weight of the prefrontal cortex and nucleus accumbens	(Clarke and Schneider 1993; Coe et al. 2003; Diz-Chaves et al. 2012; Fatima et al. 2017; Frye and Wawrzycki 2003; Guan et al. 2013; Hao et al. 2010; Jia et al. 2015; Koehl et al. 1999; Lemaire et al. 2000; Lin et al. 2012; Lin and Wang 2014; Uno et al. 1990; Wakshlak and Weinstock 1990)
	Hypersensitivity to amphetamine		
	Anhedonia		
	Increased helplessness		
		Alterations in HPA axis and neurotransmitter levels in early development	
	Increased anxiety		
	Impaired cognition including working memory deficits		
		Reduced BDNF levels	
	Decreased exploratory behavior	Decreased Bcl-2 level	
		Diminished neurogenesis	
		Increased mGluR1 and mGluR2	
		Altered immune system	
		Stimulating dopaminergic transmission	
	Social withdrawal		
Postnatal stress	Hypoactivity	Hippocampal development, memory, spatial and social learning, response to stress of the HPA axis	(Caldji et al. 2000b; Duman et al. 2016; Duman and Monteggia 2006; Huot et al. 2002; Huot et al. 2001; Kalinichev et al. 2002; Ladd et al. 2000, 2004; Lippmann et al. 2007; Magariños et al. 2011; McIntosh et al. 1999; Wigger and Neumann 1999)
	Increased stereotypies		
	Increased anxiety behavior		
	Heightened response to acute stressor		
		Decreased BDNF level	
	Elevated PPI response		
		Neuronal atrophy	
		Stimulating dopaminergic transmission	
Chronic stress (through, e.g., repeated social defeat)	Depressive-like behavior	Disrupted circadian rhythms and immune function	(Berton et al. 1998; Crawford et al. 2013; Hollis et al. 2010; Hollis and Kabbaj 2014; Leuner et al. 2014; Maier and Seligman 1976; Meerlo et al. 1996; Porsolt et al. 1977; Ruis et al. 1999; Steru et al. 1985; Tidey and Miczek 1997; Tornatzky and Miczek 1993; Wulsin et al. 2016)
	Hypoactivity		
	Reduced exploration		
	Reduced aggression		
	Hyposexuality		
	Elevated anxiety		
	Submissive behavior		
	Social avoidance		
*Immune system*			
Maternal immune activation	Increased locomotor response to amphetamine	Increased inflammation	(Bakos et al. 2004; Cotter et al. 1995; Eßlinger et al. 2016; Fernández de Cossío et al. 2017; Kneeland and Fatemi 2013; Meyer et al. 2005; Remus and Dantzer 2016; Ronovsky et al. 2017; Rose et al. 2017; Shi et al. 2003; Wachholz et al. 2017; Zuckerman et al. 2003)
		Increased striatal DA release	
	Increased repetitive and stereotypic behavior		
	Increased anxiety		
	Helplessness		
	Disrupted sensorimotor gating		
	Impaired working memory		

### Sensitization models and neurotransmitters in general

Nearly 100 years ago, Kraepelin made the observation that with an increasing number of episodes the course of the illness worsens, the so-called sensitization model of BD (Kraepelin 1909; Post 1992). Later on behavioral sensitization to psychostimulants in rodents was used to resemble the shortening of interepisodic intervals during the progression of BD in humans (Post 1990). Repeated administration of the same dose of cocaine induced hyperactivity and elevated stereotypy responses in rats (Kilbey and Ellinwood 1977; Post 1990). A drug-high state after the administration of psychostimulants was furthermore associated with increased aggression (Borison et al. 1978; Davies et al. 1974), a declined cognitive performance (Fries et al. 2015; Rygula et al. 2015) and deficits in prepulse inhibition (PPI) (Zheng et al. 2013). Several different psychostimulants were administrated to induce mania-like behavior. Apart from cocaine, the used substances were amphetamine (Frey et al. 2006), lisdexamfetamine dimesylate (Macêdo et al. 2013), and fenproporex in rats (Rezin et al. 2014), and alpha-lipoid acid (Macêdo et al. 2012) and GBR12909 in mice (Queiroz et al. 2015). All these substances resulted in mania-like behavior, which could be attenuated by mood stabilizers like valproate or lithium (Sharma et al. 2016).

Withdrawal from psychostimulants is accompanied by depressive-like behavior or at least an anhedonic state measured as reduced sexual behavior (Barr et al. 1999), elevated thresholds in self-stimulation (Markou and Koob 1991; Wise and Munn 1995), reduced activity (Paulson et al. 1991), decreased sucrose consumption (Barr and Phillips 1999), increased negative contrast (Barr and Phillips 2002), and increased anxiety (Mutschler and Miczek 1998). Immobility time in the forced swim test, however, depends on the administered does of amphetamine as well as the training procedure and was reported to be reduced during amphetamine withdrawal (Schindler et al. 1994) but also to be increased (Marszalek-Grabska et al. 2016). Withdrawal furthermore induces supersensitivity of serotoninergic neurons (Baumann and Rothman 1998), a transient decrease in norepinephrine (NE) in the hypothalamus, and a reduction in responsiveness to amphetamine in terms of DA concentrations in caudate-putamen and nucleus accumbens (Paulson et al. 1991). Even a switch in behavior has been shown in the same animal model. Anhedonic symptoms after withdrawal stabilize after a while (Paulson et al. 1991; Wise and Munn 1995). Morphine pretreated rats, furthermore, show a switch in β-endorphin-induced locomotor activity from being hyper-responsive to hypo-responsive when going through withdrawl (Schwartz et al. 1982).

The administration of psychostimulants affects several neurotransmitter systems which is in line with the fact that a number of neurotransmitters are affected in patients with BD (Barchas and Altemus 1999; Schildkraut 1965). Similarly, other pathways, e.g., the phosphoinositide cycle have been reported to be involved in BD and are targeted by treatment options for the disorder (Agam et al. 2002). A comprehensive overview of all these neurotransmitters and pathways would go beyond the scope of this review; therefore, we will give a brief overview on neurotransmitters and then focus on the dopaminergic system in “Dopaminergic pathways.”

The cholinergic system seems to be predominantly involved in depressive-like symptoms in humans and animals. Manipulation of the cholinergic system through administration of arecoline, a direct agonist of cholinergic receptors, induced depression in both healthy controls and unmedicated euthymic BD patients (Nurnberger et al. 1983). Furthermore, affective disorder patients undergo exaggerated depressive responses to cholinergic agents compared to control groups. This hypersensitivity to cholinergic manipulations supports the hypothesis of a cholinergic imbalance during depressive episodes (van Enkhuizen et al. 2015b; Janowsky et al. 1994). Further evidence for the involvement of acetylcholine in BD comes from neuroimaging studies, where physostigmine, a cholinesterase inhibitor, resulted in elevated acetylcholine levels in the brain, counteracted mania, and induced depressive-like symptoms in both control and patients with affective disorders (Hannestad et al. 2013; Janowsky et al. 1972; Janowsky et al. 1974). Also reduced β_2_ nicotinic acetylcholine receptor availability was observed in depressed BD patients compared to healthy and euthymic individuals (Hannestad et al. 2013). The brains of depressed patients contain elevated levels of choline, the precursor of acetylcholine (Steingard et al. 2000), supporting a hypercholinergic state during depressive episodes (van Enkhuizen et al. 2015b).

Animal models confirm the involvement of the cholinergic system in depression. The α7 nicotinic acetylcholine receptor agonist SSR180711 shows antidepressant-like effects in mice, indicated by reduced immobility time in the forced swim test (Andreasen et al. 2012). Interestingly, behaviorally effective doses of SSR180711 inhibited in part the serotonin reuptake (Andreasen et al. 2012). Another subtype-selective nicotinic acetylcholine receptor agonist acts antidepressive-like by reversing the escape deficits in the learned helplessness paradigm in rats (Ferguson et al. 2000). Furthermore, nicotine attenuates anhedonia-like behavior in rats (Andreasen et al. 2011) and depressive-like behavior in mice (Andreasen and Redrobe 2009). The withdrawal of chronic nicotine administration induces depressive-like behavior in mice, measured as increased immobility time in the force swim test (Roni and Rahman 2014). Another manipulation of the cholinergic system is the inhibition of acetylcholinesterase, which is degrading acetylcholine. This inhibition induces depressive- and anxiety-like behavior in mice (Mineur et al. 2013). Interestingly, both lithium and valproate increase the activity of acetylcholinesterase in the brain of rats (Varela et al. 2013).

The catecholaminergic system on the other hand is mainly involved in mania-like symptoms in humans as well as in animals. Elevated levels of both DA and NE could be observed in BD rapid cycling (Juckel et al. 2000) and normal cycling patients (Berk et al. 2007). Several antidepressants increase synaptic catecholamine (Salvi et al. 2008; Tanda et al. 1994) levels. Furthermore, as already mentioned above the psychostimulant amphetamine increases synaptic DA and NE levels through inhibition or reversing the corresponding reuptake mechanisms or elevated DA efflux (Berk et al. 2007; Raiteri et al. 1975; Schaeffer et al. 1976; Sulzer et al. 2005). Amphetamine not only induces mania-like behavior in animals but also causes manic symptoms in healthy humans and BD patients, such as decreased need for sleep, elevated mood, drive and energy and attention, sleep, sexual behavior, sensorimotor function, learning and memory are affected (Asghar et al. 2003; Berk et al. 2007; Corp et al. 2014; Cousins et al. 2009; Jacobs and Silverstone 1986; Nurnberger et al. 1982; Peet and Peters 1995; Seiden et al. 1993). These behavioral effects of amphetamine administration are not only a simple consequence of increased neurotransmitter levels but it is more likely that a disturbance of the homeostatic mechanisms, controlling the catecholamine levels, plays a key role for this dramatic mood shift (Anand et al. 1999; van Enkhuizen et al. 2015b; Young and Dulcis 2015). Comparable to the animal models psychotherapeutics, such as lithium and valproate, can also attenuate the amphetamine mania-relevant behavior in humans (Flemenbaum 1974; Silverstone et al. 1980; Van Kammen and Murphy 1975).

Several neurotransmitters are affected in BD and together with the high comorbidity of BD with substance abuse disorders (Kessler et al. 1997; Regier et al. 1990; Salloum et al. 2005) sensitization models have some face validity. They have for a long time also been the only BD animal models that show both phases, mania and depression, in the same animal model (Kato et al. 2007). On the other hand, the fact that psychostimulants act on numerous pathways hampers the investigation of the neurobiology underlying the observed behavioral changes. Therefore, more recent models for BD try to focus on one neurotransmitter system, primarily the dopaminergic system. Its involvement in BD and manipulations in animal models will be discussed in “Dopaminergic pathways.”

Another limitation of the psychostimulant induced animal models for BD mania is that only a few simple aspects of human mania, like hyperactivity, are mimicked and even the amphetamine-induced hyperactivity is not specific for BD (Logan and McClung 2016; Rygula et al. 2015).

### Dopaminergic pathways

Evidence from both human and animal studies suggests that BD is caused by an impaired neurotransmitter homeostasis (Berk et al. 2007). Clinical observations revealed that DA is altered in both episodes of BD. Thus, the dopamine hypothesis, claiming that dopaminergic transmission is disturbed depending on the mood phase, is one of the most promising hypotheses for the pathophysiology of BD (Berk et al. 2007). Manic episodes are associated with hyperdopaminergia. Increased dopaminergic transmission then induces homeostatic regulation mechanisms, which in a next step downregulate the post- and presynaptic sensitivity of receptors among other mechanisms. This downregulation results in an episode of decreased dopaminergic transmission, which is associated with the depressive episode of BD. This hypodopaminergic state activates then again the same homeostatic mechanisms, which now upregulate the key elements, resulting in another manic episode and thereby explaining the cyclic nature of the disease. A desynchronization of receptors and other key elements in different brain regions might be a possible explanation for euthymic episodes (Berk et al. 2007).

The dopaminergic system is involved in experiencing pleasure, mediating motivation (Bressan and Crippa 2005), impulsivity, risk behavior, and cognitive processes (Seamans and Yang 2004). Manipulation of the dopamine D1 receptor (D1R) in several species results in working memory deficits (Floresco and Phillips 2001; Goldman-Rakic 1999; Paspalas et al. 2013; Puig et al. 2014). Furthermore, increased D1R expression in the prefrontal cortex plays a role in impulsivity (Loos et al. 2010), cocaine addiction, sucrose preference, and high-risk behavior (Sonntag et al. 2014). All these behaviors are to a certain extent detectable in patients of BD, including increased risk for substance abuse (Messer et al. 2017), increased impulsivity in manic episodes (Logan and McClung 2016), or cognitive deficits (Cope et al. 2016). Indeed, elevations in D1R have been observed in BD patients via positron emission tomography and single-photon emission computed tomography (Gonul et al. 2009; Suhara et al. 1992; Yao et al. 2013). Also other dopamine receptors are altered in BD patients and might therefore play a role in the pathogenesis of the disease. The D2R density is elevated in the nucleus accumbens in bipolar patients with psychotic symptoms (Pearlson et al. 1995). Elevated DA levels in the urine were additionally observed in BD patients within manic episodes (Joyce et al. 1995). Alterations in the dopaminergic system through administration of psychostimulants result in a shift of neurotransmitter levels and manic behavior in humans (Cousins et al. 2009). DA levels can be affected through administration of L-DOPA, the precursor of DA, which is an established medication for Parkinson’s disease (Berk et al. 2007). L-DOPA administration induced behavior similar to BD mania in these patients, such as increased sexual behavior, impulsivity, and risk taking (Berk et al. 2007; Claassen et al. 2011; van Praag and Korf 1975; Raja and Bentivoglio 2012). Not all, but some BD patients treated with different DA agonists, such as bromocriptine, also experienced manic episodes (Fisher et al. 1991; Gerner et al. 1976). BD depression can be treated with such agonists and resulted in an improvement of depressive symptoms (Goldberg et al. 2004; Zarate et al. 2004). On the other hand, manic symptoms of BD patients can be attenuated through administration of DA antagonists (Christie et al. 1989; Tohen et al. 2003; Vieta et al. 2005). Furthermore, antidepressants increase the dopaminergic transmission in the prefrontal cortex and nucleus accumbens as shown in rodents (Tanda et al. 1994). Alterations of functional DA transporter (DAT) levels, particularly reduced availability, were confirmed in BD patients via positron emission tomography (Anand et al. 2011), in postmortem tissue (Rao et al. 2012; Young and Dulcis 2015) and cell culture experiments (Horschitz et al. 2005).

A knock-down of DAT in mice with reduced DAT functioning to approximately 10% of wild-type level resulted in mania-like behavior, such as hyperactivity in novel environments (Zhuang et al. 2001), increased risk behavior in the Iowa Gambling Task (IGT) (Young et al. 2011), impaired decision making with a preference for high reward combined with high risk in the IGT (van Enkhuizen et al. 2014b), and a similar hyperexploratory behavior in the BPM as observed in humans but with less straight movements compared to BD patients (van Enkhuizen et al. 2014a; Perry et al. 2009; Young et al. 2010). But DAT knock-down mice fail to mimic the observed sensorimotor deficits in PPI observed in humans (Ralph-Williams et al. 2003). Alpha-methyl-*p*-tyrosine induced depletion of DA and was able to attenuate some of the behavioral abnormalities of the DAT knock-down mice (van Enkhuizen et al. 2014a), similar to the effect of chronic valproate treatment (van Enkhuizen et al. 2013a), whereas both treatments did not affect the exploration behavior. To sum up, this animal model is able to resemble various behavioral aspects of human BD mania (Cassidy et al. 1998; van Enkhuizen et al. 2015a). Interestingly, photoperiod length can influence the DAT level in the brain of rats (Dulcis et al. 2013), thereby again connecting the dopaminergic system and the circadian rhythm (see “Circadian rhythm”) with the pathophysiology of BD. Possible critiques of the DAT knock-down mice are the clearly too low *DAT* expression compared with unmedicated BD patients, which is approximately 80% of healthy subjects (Anand et al. 2011; Young and Dulcis 2015) and that this animal model mimics only BD mania and not depression (van Enkhuizen et al. 2015a). Mania-like behavior, such as hyperactivity (Giros et al. 1996; Ralph et al. 2001) and sensorimotor deficits in PPI (Ralph et al. 2001), can also be induced through a complete knock-out of the *DAT* gene in mice. The pointed hyperactivity of these mice could be attenuated through the D1R antagonist SCH23390 (Ralph et al. 2001). PPI deficits could be diminished through clozapine and quetiapine, which are atypical antipsychotics used as an effective treatment for BD mania and schizophrenia (Powell et al. 2008), acting as antagonists of the D2R (Brust et al. 2015; Masri et al. 2008). However, the DAT knock-down and knock-out mice were also used as animal models for ADHD (Leo and Gainetdinov 2013) and schizophrenia (Gainetdinov et al. 2001). This is no surprise considering the fact that DA transmission is also involved in the pathogenesis of these disorders (Gainetdinov et al. 2001; Giros et al. 1996; Leo and Gainetdinov 2013; Sharma et al. 2016; Zhuang et al. 2001). Nevertheless, we want to point out that it is important to create specific animal models, which are able to model more facets of the complex behavior of each of these disorders and the cyclical nature of BD especially.

One different approach to recreate the cyclical nature of BD within one animal through manipulating the dopaminergic system was realized by Freund and colleagues, using an inducible lentiviral vector system. Over-expression of D1R in the medial prefrontal cortex of rats resulted in increased sexual behavior, increased sucrose preference, impulsivity, and increased drug-related behavior (Sonntag et al. 2014). The increased D1R expression in the medial prefrontal cortex was accompanied by decreased D2R expression in the nucleus accumbens. Even more interestingly, just the termination of this viral over-expression was sufficient enough to induce depressive-like behavior in the triadic paradigm of learned helplessness, reduced activity, and diminished sucrose preference (Freund et al. 2016). Termination of D1R over-expression furthermore increased levels of cAMP response element-binding protein (CREB) in nucleus accumbens (Freund et al. 2016). This animal model is therewith one of the few models, which is able to recreate the cyclical nature of BD. It furthermore supports the hypothesis that the homeostatic regulation of DA transmission plays a key role in the pathogenesis of BD (Berk et al. 2007).

Animal models that manipulate dopaminergic pathways mainly increased dopaminergic transmission and therewith induced mania-like behavior (Table 1). Inducible viral over-expression of D1R allowed investigating behavior after the termination of the over-expression (Freund et al. 2016). Results indicate that while increased dopaminergic transmission is associated with mania-like behavior the termination of increased dopaminergic transmission induces depressive-like behavior. Exact mechanisms leading to the observed behavioral changes are still unclear. Autoregulatory mechanism might have downregulated D1R expression during the viral over-expression causing reduced dopaminergic transmission after the termination of the over-expression. This explanation would be in line with Berk’s dopamine hypothesis of BD (Berk et al. 2007). While this approach might be a promising new way to create an animal model for BD, further studies, e.g., on the models’ susceptibility to treatment like lithium are necessary for better understanding of the underlying mechanisms.

### Environment: stressors

Stressful life events in combination with genetic factors are a risk factor for the onset of psychiatric disorders (Afifi et al. 2009; Bebbington et al. 1984; Costello 1982; Kendler et al. 1999; Paykel 1978; Schmitt et al. 2014; Surtees et al. 1986). Environmental risk factors, causing these stressful experiences, are for example neglect during childhood, maternal loss, economic problems, family violence, abuse, sexual maltreatment, and many more (Bernstein et al. 2003; Brown et al. 2009; Kaufman and Charney 2001; Marangoni et al. 2016; Mullen et al. 1996). In fact almost two-third of BD patients sustained at least one negative or goal-attainment life event 6 months prior to the index or first occurred episode (Simhandl et al. 2015). Especially the first period of life plays an important role in the development of children (Vetulani 2013). Different types of maltreatment including physical, sexual, and emotional abuse or neglect in the early period of life (i.e., early live stress, ELS) are associated with mood disorders (Afifi et al. 2009; Green et al. 2010; Hovens et al. 2010; Leverich et al. 2002; McLaughlin et al. 2010). ELS in humans can lead to impaired cognitive functioning, exemplified in worse academic performance, impaired intellectual ability, language difficulties, and lower IQ (Cohen et al. 2008; De Bellis et al. 2009; van den Dries et al. 2010; Loman et al. 2009; Nelson et al. 2007). The risk to develop anxiety, depression, and psychoses in adulthood is particularly increased through ELS (Bebbington et al. 2004; Gilbert et al. 2009; Kaufman and Charney 2001; Mullen et al. 1996) and it is more likely that these illnesses are treatment resistant (Bryer et al. 1987; Nemeroff et al. 2003; Vetulani 2013). These long-lasting effects can occur through high or chronic levels of stress, because they might affect brain development and thereby mental health (Anda et al. 2006; De Bellis et al. 1999a; De Bellis et al. 1999b; Lupien et al. 2009; Maniglio 2009; McLaughlin et al. 2010; Pechtel and Pizzagalli 2011; Pirkola et al. 2005; Spataro et al. 2004; Teicher 2002). 32 percent of psychiatric disorders can be explained by childhood adverse experiences (Green et al. 2010; Pechtel and Pizzagalli 2011). These experiences influence also the overall lifespan, because humans, who experienced more than six traumatic events in their childhood, have an increased risk of dying approximately 20 years earlier (Anda et al. 2009; Brown et al. 2009).

The mother–infant interaction is very important not only for humans, but also for primates, rodents, or mammals in general for the development of the offspring and includes much more than just supply with nutrition (Gutman and Nemeroff 2002; Harlow and Zimmermann 1959; Heim and Nemeroff 2002; Kaffman and Meaney 2007; Mason and Berkson 1975; Meaney 2001; Tractenberg et al. 2016). Stressful events in the early period of life produce long-lasting effects on brain development in rodents (Caldji et al. 1998, 2000a; Francis and Meaney 1999; Romeo et al. 2003; Tractenberg et al. 2016), comparable to the effects observed in humans.

ELS in rodents can be induced as early as the prenatal period by stressing the pregnant damn, e.g., by restrain. Observed behavioral consequences of adult rats, which were prenatally stressed, can be identified as depressive-like, namely anhedonia, increased helplessness, indicated through increased immobility time in the forced swim test, increased anxiety behavior in the open field test, impaired cognition, decreased exploratory behavior, and social withdrawal (Fatima et al. 2017; Frye and Wawrzycki 2003; Hao et al. 2010; Jia et al. 2015; Lin et al. 2012; Lin and Wang 2014; Wakshlak and Weinstock 1990). Similar long-lasting effects on social behavior could be observed in prenatally stressed rhesus macaques (Clarke and Schneider 1993).

Prenatal stress is associated with alterations in the immune system (Diz-Chaves et al. 2012, see “Immune system”) and HPA axis (Koehl et al. 1999), decreased neurogenesis in the hippocampus (Fatima et al. 2017; Lemaire et al. 2000; Lin and Wang 2014), and reduced BDNF levels (Jia et al. 2015; Lin and Wang 2014). In addition, the metabotropic glutamate receptor 1 (mGluR1) is increased in the hippocampus and prefrontal cortex and mGluR5 is increased in the striatum of prenatally stressed rats (Jia et al. 2015). Glutamate transporter expression is increased in adult prenatally stressed rats and therefore affects the whole glutamate neurotransmission long term, but only in the frontal cortex and hippocampus (Adrover et al. 2015). The glutamatergic system plays a critical role in the regulation of synaptic plasticity (D’Sa and Duman 2002; Manji et al. 2001). Chronic lithium and valproate treatment can downregulate the synaptic expression of ionotropic glutamate receptors in hippocampal neurons of rats (Du et al. 2004; Gray et al. 2003) and the antidepressant imipramine has the opposite effect (Einat and Manji 2006; Gray et al. 2003). In addition, pharmacological inhibition of ionotropic glutamate receptors via several competitive and noncompetitive inhibitors attenuated amphetamine-induced hyperactivity in mice (Vanover 1998). Interestingly, a contrary effect occurs through manipulating the GluR6. Knock-out mice for the *GluR6* gene exhibited mania-like behavior, indicated through hyperactivity, heightened responsivity to amphetamine, increased risk-taking behavior, more aggressive, less immobility time in the forced swim test, and less anxiety behavior (Shaltiel et al. 2008). Lithium was able to attenuate these behavioral abnormalities (Shaltiel et al. 2008). Prenatal stress is in addition able to alter the circadian rhythm and corticosterone secretion through disturbance of the HPA axis in adult prenatally stressed rats (Koehl et al. 1999). One function of the HPA axis is the synthesis of cortisol, a glucocorticoid; especially, maternal glucocorticoid is crucial for fetal development, because it affects synaptic connections, density, and differentiation of postnatal development of the fetal hippocampus (Fatima et al. 2017; Trejo et al. 2000). An overall decrease in the number of neurons and overall size could be observed in fetal hippocampus of rhesus macaques, whose mothers were administrated excessive amounts of glucocorticoids during pregnancy (Uno et al. 1990). Similar effects of inhibited hippocampal neurogenesis, decreased hippocampal volume, and elevated cortisol levels were observed in juvenile rhesus macaques prenatally stressed via environmental alterations of the photoperiod (Coe et al. 2003). Hippocampal *Bcl*-*2* expression is also affected, namely decreased, in juvenile prenatally stressed offspring rats, exhibiting depressive-like behavior (Guan et al. 2013). Interestingly lithium, which attenuates mania-like behavior in animal models, increases the Bcl-2 level (Chen et al. 1999; Manji et al. 2000), indicating one way of lithium’s therapeutic effects. In return, chronic stress during pregnancy affects not only the unborn pup, but also the mother. Stressed mother rats exhibit depressive-like behavior in the forced swim test, less maternal care, and a decrease in spine density in the medial prefrontal cortex (Leuner et al. 2014).

Not only prenatal but also postnatal stress, for example, induced through maternal separation of the pups from their mother for a defined amount of time (e.g., 2–4 h per day for up to 3 weeks) can result in long-lasting behavioral alterations. Early maternal separation (EMS) results in ELS and induces a conserved neural response, including a protest response and the feeling of despair in humans and animals (Hofer 2005). The mother’s behavior has an influence on the brain development of the neonate. Hippocampal development, memory, spatial and social learning, and the response to stress of the HPA axis is affected by maternal licking and grooming of the offspring (Lévy et al. 2003; Lippmann et al. 2007; Liu et al. 1997, 2000; Vetulani 2013). This mother infant interaction has as well a physiological effect in rats and an interruption can result in alterations of the heart rate, hormone levels, and interestingly the circadian rhythm (Hofer 1970, 1975, 1976; Kuhn et al. 1990; Meaney et al. 1991; Stahl et al. 2002; Stanton and Levine 1990; Vetulani 2013). Rats exposed to chronic EMS exhibited hypoactivity, increased stereotypic behavior, abnormal anxiety behavior, abnormal HPA axis functioning, and heightened response to an acute stressor and an elevated acoustic startle response in adulthood (Caldji et al. 2000b; Huot et al. 2001; Kalinichev et al. 2002; Ladd et al. 2000, 2004; Lippmann et al. 2007; McIntosh et al. 1999; Wigger and Neumann 1999). Antidepressant treatment can attenuate these depressive-like behaviors induced by EMS (Cotella et al. 2013; Couto et al. 2012; El Khoury et al. 2006; Huot et al. 2001; MacQueen et al. 2003). The BDNF levels were decreased over a long period of time in the hippocampus of adult rats exposed to EMS (Lippmann et al. 2007), as well as in rats that were chronically stressed (Smith et al. 1995). Prolonged stress experiments indicate that inhibition of BDNF could cause neuronal atrophy or that BDNF is required for neuronal remodeling (Duman et al. 2016; Magariños et al. 2011). Indeed, long-lasting reduced BDNF levels in the hippocampus were associated with learning and memory deficits, as well as depressive-like behavior (Duman and Monteggia 2006; Huot et al. 2002; Lippmann et al. 2007). Furthermore, *BDNF* expression can be increased through chronic antidepressant treatment in rat hippocampus (Nibuya et al. 1995; Russo-Neustadt et al. 2000). In return, a single direct injection of BDNF into the hippocampus led to an antidepressant effect, indicated through comparable performances in the forced swim test and learned helplessness paradigm of rats chronically treated with antidepressants (Shirayama et al. 2002). This behavioral effect could not be recapitulated in mice with reduced *BDNF* expression (Saarelainen et al. 2003), suggesting that BDNF signaling is necessary for the antidepressant effect.

Interestingly, stress affects the dopaminergic system through stimulating dopaminergic transmission in the VTA of stressed rats (Di Chiara et al. 1999; Horger and Roth 1996; Nieoullon and Coquerel 2003; Yadid et al. 2001), which again links alterations in the dopaminergic system and stress to BD. In addition, humans and rodents are not only in these early stages of life highly vulnerable to stress, but also in adolescence. During this important time of brain development, neuroplasticity in stress regulatory circuits and HPA axis functioning are formed (Andersen 2003; Andersen and Teicher 2008; Eiland and Romeo 2013; Wulsin et al. 2016). Therefore, chronic stress exposure to rats in the late adolescence results in depressive-like behavior in adulthood (Wulsin et al. 2016). But the effects of environmental stressors can be so powerful to induce depressive-like phenotypes even in adult, normally raised rats. These stressors can be provided through several behavioral paradigms, such as the forced swim test (Porsolt et al. 1977), tail suspension test (Steru et al. 1985), and learned helplessness (Maier and Seligman 1976). Similar behavioral abnormalities, which can be described as depressive-like behavior, such as reduced locomotor and exploratory behavior, aggression, sexual behavior, elevated anxiety and submissive behavior, social avoidance, disrupted circadian rhythms, and immune function, can be induced through repeated exposure to social defeat in rats (Berton et al. 1998; Crawford et al. 2013; Hollis et al. 2010; Hollis and Kabbaj 2014; Meerlo et al. 1996; Ruis et al. 1999; Stefanski 2000; Tidey and Miczek 1997; Tornatzky and Miczek 1993). Another prominent stressor, sleep deprivation, was already discussed in “Circadian rhythm” and can result in mania-like behavior in rodents (Malkoff-Schwartz et al. 1998).

Environmental stress has been widely used to create animal models for depression. Paradigms like maternal separation (Tractenberg et al. 2016) or social defeat (Toyoda 2017) are well-established models that have revealed several neurobiological mechanisms that connect stress and the onset of depression. It is to mention, however, that we cannot distinguish between uni- and bipolar depressive-like behavior in these models. The administration of antidepressants in these models provides mixed results (Harrison and Baune 2014) but to our knowledge it did not result in the induction of mania-like behavior as sometimes seen in patients with BD. Lithium was able to prevent ELS-associated changes in the brain (Husum and Mathé 2002).

Environmental stressors can also induce mania-like behavior, e.g., by affecting circadian rhythms (Malkoff-Schwartz et al. 1998). A combination of circadian rhythm manipulation and environmental stressors (comparable to Le-Niculescu et al. 2008; see “Circadian rhythm”) might therefore be useful to create a switch between both behavioral phases in animal models.

### Immune system

During the past few years, it became more and more evident that the immune system plays an important role in psychiatric disorders including BD. First speculations started after epidemiological studies revealed that BD occurs more often in people born between December and March (Fuller Torrey et al. 1996), indicating that an infection of the mother during the winter months could contribute to the risk to develop BD. Indeed, several years later it was confirmed that an influenza infection during pregnancy increases the risk for the offspring to develop BD by fourfold (Parboosing et al. 2013). BD patients furthermore show an increased cerebrospinal fluid-to-serum ratio, which could be an indicator for a dysfunctional blood–brain barrier (Patel and Frey 2015; Zetterberg et al. 2014). However, not only the immune system predisposed to develop BD, but also changes in the immune system have been shown in patients diagnosed with BD. A persistent and low-grade pro-inflammatory state, which is more intense during mood episodes, especially manic episodes, and less intense in depressive episodes (Brietzke et al. 2009b; Modabbernia et al. 2013) has been revealed in these patients. Even euthymia has been associated with detectable peripheral pro-inflammatory activity (Brietzke et al. 2009a, 2009b). In support of these findings is the increased mortality rate of BD patients (Anda et al. 2009; Brown et al. 2009; Crump et al. 2013). Apart from suicide, this elevated mortality rate can be explained by additional natural causes of death associated with increased inflammation (Crump et al. 2013; Hoang et al. 2011; Kupfer 2005). Furthermore, the immunological response to stress is altered in patients with BD (Wieck et al. 2014). There are some findings showing that the number of past episodes could even be a key factor to understand the evolution of immunological changes in BD. In a study conducted by Maes and colleagues, they have found that the number of past depressive episodes positively correlates with pro-inflammatory cytokines (Maes et al. 2012). Indeed, elevated levels of pro-inflammatory cytokines were reported in BD patients (Goldstein et al. 2009; Haarman et al. 2014; Stertz et al. 2013). Interestingly, lithium is able to attenuate pro-inflammatory cytokines (Boufidou et al. 2004; Green and Nolan 2012; Himmerich et al. 2013; Patel and Frey 2015; Rowse et al. 2012; Wang et al. 2009; Zhang et al. 2009).

In animal models, maternal immune activation (MIA) and its consequences for the offspring has been intensively studied in the last two decades (Meyer 2014). Thereby, the immune system of the pregnant damn has mainly been stimulated with the human influenza virus (Cotter et al. 1995; Kneeland and Fatemi 2013), the immunostimulant Polyinosinic:polycytidylic acid (poly I:C) (Eßlinger et al. 2016; Rose et al. 2017; Shi et al. 2003), or bacterial lipopolysaccharide (Bakos et al. 2004; Fernández de Cossío et al. 2017) during different gestational stages. In adult animals that had been exposed to MIA sensorimotor gating, i.e., latent inhibition and the US-preexposure effect are disrupted, impairments in working memory are evident and the locomotor response to amphetamine is increased (Meyer et al. 2005). Furthermore, a reduction in social interactions in addition to increased repetitive and stereotypic behavior has been reported (Fernández de Cossío et al. 2017; Rose et al. 2017). Even depressive-like behavior including increased anxiety and helplessness (Meyer et al. 2005; Ronovsky et al. 2016) has been shown. Thereby, depressive-like behavior has also been reported in the second generation after MIA (Ronovsky et al. 2017). Taken together, MIA results in the development of several behavioral deficits that are associated with psychiatric disorders. Given the strong effect on sensorimotor gating and deficits in social behavior, MIA in animal models has mainly been associated with a schizophrenia-like phenotype (Meyer et al. 2005) or autism-related characteristics (Fernández de Cossío et al. 2017). An animal model for BD using MIA has never been proposed. At the same time, deficits in sensory gating have been implicated in patients with BD (Cheng et al. 2016), specifically during the acute mania phase (Kohl et al. 2013). Similarly, impairments in working memory (Dickinson et al. 2017) and social cognition (Hoertnagl et al. 2014) have been reported in patients with BD and animals after MIA (Fernández de Cossío et al. 2017; Meyer et al. 2005). Further findings on an increased striatal DA release following MIA (Zuckerman et al. 2003) are in line with the hypothesis that dopaminergic pathways are disrupted in BD (see “Dopaminergic pathways”) and further support the fact that MIA might be useful to also create animal models for BD. Less research on disrupted behavior after an acute or chronic activation of the immune system in adult animals has been conducted. Nevertheless, there is evidence that even in adulthood activation of the immune system can induce depressive-like behavior and anxiety (Remus and Dantzer 2016; Wachholz et al. 2017). So far no reports on adult immune activation and mania-associated behaviors in animal models exist. An animal model of mania (induced by amphetamine; see “Sensitization models and neurotransmitters in general”), however, showed increased cytokine levels in plasma and brain, which could together with the mania-like behavior be reversed by lithium treatment (Valvassori et al. 2015). Even adult animals therefore show depressive-like behavior when manipulating the immune system and a mania-like phenotype in adult animals is associated with increased inflammation.

Taken together, there is growing evidence that the immune system plays an important role in BD. Animal models with an immune activation early in development (i.e., prenatally) show several behavioral changes that are associated with depression and mania. So far, however, no cycling between these two behavioral states has been shown after manipulation of the immune system and MIA has never been considered as a manipulation to create an animal model for BD.

## Conclusions

BD was to some extent already described by ancient Greek scholars like Hippocrates and Aristoteles (Angst and Marneros 2001). Nevertheless, our current knowledge in the 21st century about the disorder is still limited. Given the fact that lithium, one of the top choice treatment options (Sani et al. 2017), was discovered by animal research (Cade 1949), animal models for the disorder can be a very useful tool to advance our knowledge. Indeed, several models have either confirmed findings from patients or even extended these findings by explaining the underlying neurobiological mechanisms. In this review, we could confirm that several affected areas and risk factors can be found in human patients as well as animal models for BD. Thereby, it can be noticed that all sections described here are connected with each other. Disruptions of circadian rhythms influence the dopaminergic system (Coque et al. 2011; McClung et al. 2005; Mukherjee et al. 2010; Roybal et al. 2007). DA in turn is well known as a key player in reward behavior and therewith drug use (Cooper et al. 2017). Substance abuse disorder is correlated with a stressful environment and stress increases the risk for relapse (Goldstein et al. 2008). Furthermore, stress and addiction induce similar epigenetic and neurobiological modifications (Cadet 2016; Moonat and Pandey 2012; Palmisano and Pandey 2017; Spanagel et al. 2014). Stress has an influence on the immune system (Stefanski 2000) and the immune system on the other hand is connected with pathways related to circadian rhythm (Dumbell et al. 2016). Manipulation in animal models therewith often results in an interplay of several affected factors. It therewith resembles the human condition and it strengthens the model. At the same time, when several mechanisms are affected, the exact mechanism underlying BD is hard to investigate. It is clear that BD is not caused by a single factor. Therefore animal models that cover a broad range of the disruptions observed in human patients might need manipulations in several areas. At the same time, single-factor manipulations would be the best way to confirm correlations between observed changes and the manipulation. Due to the complex etiology, biology and disease pattern of psychiatric diseases, animal models with targeted mutations involve some limitations. Therefore, it is unlikely that one genetic alteration within an animal model will be able to recapitulate all facets of human DSM-defined disorder symptomatology (Kaiser and Feng 2015). DSM-defined disorders often contain similar phenotypical features, which can be based on diverse biological factors. Nevertheless, most established animal models for BD use one manipulation to model just a partial list of symptoms (concentrating on either mania or depressive-like symptoms). This approach facilitates to connect behavioral outcomes to specific pathways and is an important tool to advance our knowledge of certain aspects. Nevertheless, the diverse character of behavior and even switch of behavior observed in patients is not considered in these models. Specifically for the investigation of this switch and its neurobiology, it is crucial to create animal models presenting mania- as well as depressive-like behavior (Nestler and Hyman 2010). So far, very few models were able to show a switch between mania- and depressive-like behaviors. Sensitization models show mania-like behavior following repeated administration of psychostimulants (see “Sensitization models and neurotransmitters in general”) and depressive-like behavior during withdrawal (Antelman et al. 1998; Barr et al. 1999; Baumann and Rothman 1998; Marszalek-Grabska et al. 2016; Persico et al. 1995; Schwartz et al. 1982). However, psychostimulants target several neurotransmitter systems and therefore information on the neurobiology of BD we got from these models is limited. New techniques, namely the use of viral vector to induce genetic material into specific cells, might be promising for the development of new BD animal models reproducing the cyclic character of the disorder. D1R over-expression on glutamatergic cells in the medial prefrontal cortex induces mania-like behavior, while the single termination of this over-expression was sufficient enough to result in depressive-like behavior. It has to be noted, however, that this switch in behavior is not spontaneous as reported in BD patients. Apart from manipulating the dopaminergic system, circadian rhythms and the immune system seem to be promising targets for the development of animal models for BD. Investigating the neurobiology behind the induction of mania- or depressive-like behavior by extending or shortening the length of day might provide us with the switch between the two phases and will induce both phases one after the other in one animal. Similarly, inducing an increased state of inflammation followed by a decreased state of inflammation might reveal an animal model for BD showing the characteristic switch between mood phases.

As always, further research is needed. New technology, however, either in the field of animal research as well as for examining human patients is being established. Combining these techniques with new insights especially in the fields of immunology and circadian rhythms provides us with new tools to develop better models.

## Abbreviations

ADHD: attention-deficit hyperactivity disorder
Bcl-2: B-cell lymphoma 2
BD: bipolar disorder
BDNF: brain-derived neurotrophic factor
BPM: behavioral pattern monitor
Clock: circadian locomotor output cycles kaput
CREB: cAMP response element-binding protein
D1R: dopamine D1 receptor
D2R: dopamine D2 receptor
DA: dopamine
DAT: dopamine transporter
ELS: early life stress
EMS: early maternal separation
ERK: extracellular-signal-regulated kinase
GSK-3β: glycogen synthase kinase-3 beta
HPA: hypothalamic–pituitary–adrenal
IGT: Iowa Gambling Task
mGluR: metabotropic glutamate receptor
MIA: maternal immune activation
NE: norepinephrine
poly I: C: polyinosinic:polycytidylic acid
PPI: prepulse inhibition
REV-ERBα: nuclear receptor subfamily1, group D, member1
VTA: ventral tegmental area

## References

[CR1] Abulseoud OA, Camsari UM, Ruby CL, Mohamed K, Abdel Gawad NM, Kasasbeh A, et al. Lateral hypothalamic kindling induces manic-like behavior in rats: a novel animal model. Int J Bipolar Disord. 2014;2. http://www.ncbi.nlm.nih.gov/pmc/articles/PMC4452639/. Accessed 11 May 2017. 10.1186/s40345-014-0007-8 PMC4452639 26092394

[CR2] Abulseoud OA, Gawad NA, Mohamed K, Vadnie C, Camsari UM, Karpyak V (2015). Sex differences in mania phenotype and ethanol consumption in the lateral hypothalamic kindled rat model. Transl Psychiatry.

[CR3] Adrover E, Pallarés ME, Baier CJ, Monteleone MC, Giuliani FA, Waagepetersen HS (2015). Glutamate neurotransmission is affected in prenatally stressed offspring. Neurochem Int.

[CR4] Afifi TO, Boman J, Fleisher W, Sareen J (2009). The relationship between child abuse, parental divorce, and lifetime mental disorders and suicidality in a nationally representative adult sample. Child Abuse Negl.

[CR5] Agam G, Azab AN (2016). Whether lithium inhibits glycogen synthase kinase (GSK)-3β activity in vivo in humans is still an open question. Bipolar Disord.

[CR6] Agam G, Shamir A, Shaltiel G, Greenberg ML (2002). Myo-inositol-1-phosphate (MIP) synthase: a possible new target for antibipolar drugs. Bipolar Disord.

[CR7] Akhtar RS, Ness JM, Roth KA (2004). Bcl-2 family regulation of neuronal development and neurodegeneration. Biochim Biophys Acta BBA-Mol Cell Res.

[CR8] American Psychiatric Association (2013). Diagnostic and statistical manual of mental disorders.

[CR9] Anand A, Darnell A, Miller HL, Berman RM, Cappiello A, Oren DA (1999). Effect of catecholamine depletion on lithium-induced long-term remission of bipolar disorder. Biol Psychiatry.

[CR10] Anand A, Barkay G, Dzemidzic M, Albrecht D, Karne H, Zheng Q-H (2011). Striatal dopamine transporter availability in unmedicated bipolar disorder. Bipolar Disord.

[CR11] Anda RF, Felitti VJ, Bremner JD, Walker JD, Whitfield C, Perry BD (2006). The enduring effects of abuse and related adverse experiences in childhood. Eur Arch Psychiatry Clin Neurosci.

[CR12] Anda RF, Dong M, Brown DW, Felitti VJ, Giles WH, Perry GS (2009). The relationship of adverse childhood experiences to a history of premature death of family members. BMC Public Health.

[CR13] Andersen SL (2003). Trajectories of brain development: point of vulnerability or window of opportunity?. Neurosci Biobehav Rev.

[CR14] Andersen SL, Teicher MH (2008). Stress, sensitive periods and maturational events in adolescent depression. Trends Neurosci.

[CR15] Anderson IM, Haddad PM, Scott J (2012). Bipolar disorder. BMJ.

[CR16] Andreasen JT, Redrobe JP (2009). Antidepressant-like effects of nicotine and mecamylamine in the mouse forced swim and tail suspension tests: role of strain, test and sex. Behav Pharmacol.

[CR17] Andreasen JT, Henningsen K, Bate S, Christiansen S, Wiborg O (2011). Nicotine reverses anhedonic-like response and cognitive impairment in the rat chronic mild stress model of depression: comparison with sertraline. J Psychopharmacol Oxf Engl.

[CR18] Andreasen JT, Redrobe JP, Nielsen EØ (2012). Combined α7 nicotinic acetylcholine receptor agonism and partial serotonin transporter inhibition produce antidepressant-like effects in the mouse forced swim and tail suspension tests: a comparison of SSR180711 and PNU-282987. Pharmacol Biochem Behav.

[CR19] Angst J, Marneros A (2001). Bipolarity from ancient to modern times: conception, birth and rebirth. J Affect Disord.

[CR20] Antelman SM, Caggiula AR, Kucinski BJ, Fowler H, Gershon S, Edwards DJ (1998). The effects of lithium on a potential cycling model of bipolar disorder. Prog Neuropsychopharmacol Biol Psychiatry.

[CR21] Asghar SJ, Tanay VAMI, Baker GB, Greenshaw A, Silverstone PH (2003). Relationship of plasma amphetamine levels to physiological, subjective, cognitive and biochemical measures in healthy volunteers. Hum Psychopharmacol.

[CR22] Ashkenazy T, Einat H, Kronfeld-Schor N (2009). Effects of bright light treatment on depression- and anxiety-like behaviors of diurnal rodents maintained on a short daylight schedule. Behav Brain Res.

[CR23] Bakos J, Duncko R, Makatsori A, Pirnik Z, Kiss A, Jezova D (2004). Prenatal immune challenge affects growth, behavior, and brain dopamine in offspring. Ann NY Acad Sci.

[CR24] Barchas JD, Altemus M. Monoamine hypotheses of mood disorders. 1999. https://www.ncbi.nlm.nih.gov/books/NBK28257/. Accessed 22 May 2017.

[CR25] Barr AM, Phillips AG (1999). Withdrawal following repeated exposure to d-amphetamine decreases responding for a sucrose solution as measured by a progressive ratio schedule of reinforcement. Psychopharmacology.

[CR26] Barr AM, Phillips AG (2002). Increased successive negative contrast in rats withdrawn from an escalating-dose schedule of d-amphetamine. Pharmacol Biochem Behav.

[CR27] Barr AM, Fiorino DF, Phillips AG (1999). Effects of withdrawal from an escalating dose schedule of d-amphetamine on sexual behavior in the male rat. Pharmacol Biochem Behav.

[CR28] Baumann MH, Rothman RB (1998). Alterations in serotonergic responsiveness during cocaine withdrawal in rats: similarities to major depression in humans. Biol Psychiatry.

[CR29] Bebbington PE, Sturt E, Tennant C, Hurry J (1984). Misfortune and resilience: a community study of women. Psychol Med.

[CR30] Bebbington PE, Bhugra D, Brugha T, Singleton N, Farrell M, Jenkins R (2004). Psychosis, victimisation and childhood disadvantage: evidence from the second British national survey of psychiatric morbidity. Br J Psychiatry J Ment Sci.

[CR31] Bellet MM, Sassone-Corsi P (2010). Mammalian circadian clock and metabolism—the epigenetic link. J Cell Sci.

[CR32] Benedetti F, Serretti A, Colombo C, Barbini B, Lorenzi C, Campori E (2003). Influence of CLOCK gene polymorphism on circadian mood fluctuation and illness recurrence in bipolar depression. Am J Med Genet Part B Neuropsychiatr Genet.

[CR33] Benedetti F, Fresi F, Maccioni P, Smeraldi E (2008). Behavioural sensitization to repeated sleep deprivation in a mice model of mania. Behav Brain Res.

[CR34] Berk M, Dodd S, Kauer-Sant’Anna M, Malhi GS, Bourin M, Kapczinski F (2007). Dopamine dysregulation syndrome: implications for a dopamine hypothesis of bipolar disorder. Acta Psychiatr Scand.

[CR35] Bernstein DP, Stein JA, Newcomb MD, Walker E, Pogge D, Ahluvalia T (2003). Development and validation of a brief screening version of the childhood trauma questionnaire. Child Abuse Negl.

[CR36] Berton O, Aguerre S, Sarrieau A, Mormede P, Chaouloff F (1998). Differential effects of social stress on central serotonergic activity and emotional reactivity in Lewis and spontaneously hypertensive rats. Neuroscience.

[CR37] Besing RC, Paul JR, Hablitz LM, Rogers CO, Johnson RL, Young ME (2015). Circadian rhythmicity of active GSK3 isoforms modulates molecular clock gene rhythms in the suprachiasmatic nucleus. J Biol Rhythm.

[CR38] Bilu C, Einat H, Kronfeld-Schor N (2016). Utilization of diurnal rodents in the research of depression. Drug Dev Res.

[CR39] Bold RJ, Hess KR, Scott Pearson A, Grau AM, Sinicrope FA, Jennings M (1999). Prognostic factors in resectable pancreatic cancer: p53 and Bcl-2. J Gastrointest Surg.

[CR40] Bonfanti L, Strettoi E, Chierzi S, Cenni MC, Liu X-H, Martinou J-C (1996). Protection of retinal ganglion cells from natural and axotomy-induced cell death in neonatal transgenic mice overexpressing bcl-2. J Neurosci.

[CR41] Borison RL, Sabelli HC, Maple PJ, Havdala HS, Diamond BI (1978). Lithium prevention of amphetamine-induced “manic” excitement and of reserpine-induced “depression” in mice: possible role of 2-phenylethylamine. Psychopharmacology.

[CR42] Boufidou F, Nikolaou C, Alevizos B, Liappas IA, Christodoulou GN (2004). Cytokine production in bipolar affective disorder patients under lithium treatment. J Affect Disord.

[CR43] Bressan RA, Crippa JA (2005). The role of dopamine in reward and pleasure behaviour–review of data from preclinical research. Acta Psychiatr Scand Suppl.

[CR44] Brietzke E, Kauer-Sant’anna M, Teixeira AL, Kapczinski F (2009). Abnormalities in serum chemokine levels in euthymic patients with bipolar disorder. Brain Behav Immun.

[CR45] Brietzke E, Stertz L, Fernandes BS, Kauer-Sant’Anna M, Mascarenhas M, Vargas AE (2009). Comparison of cytokine levels in depressed, manic and euthymic patients with bipolar disorder. J Affect Disord.

[CR46] Brown DW, Anda RF, Tiemeier H, Felitti VJ, Edwards VJ, Croft JB (2009). Adverse childhood experiences and the risk of premature mortality. Am J Prev Med.

[CR47] Brust TF, Hayes MP, Roman DL, Watts VJ (2015). New functional activity of aripiprazole revealed: robust antagonism of D2 dopamine receptor-stimulated Gβγ signaling. Biochem Pharmacol.

[CR48] Bryer JB, Nelson BA, Miller JB, Krol PA (1987). Childhood sexual and physical abuse as factors in adult psychiatric illness. Am J Psychiatry.

[CR49] Bunney WE, Bunney BG (2000). Molecular clock genes in man and lower animals: possible implications for circadian abnormalities in depression. Neuropsychopharmacology.

[CR50] Bunney JN, Potkin SG (2008). Circadian abnormalities, molecular clock genes and chronobiological treatments in depression. Br Med Bull.

[CR51] Burdick KE, Braga RJ, Goldberg JF, Malhotra AK (2007). Cognitive dysfunction in bipolar disorder: future place of pharmacotherapy. CNS Drugs.

[CR52] Butcher GQ, Doner J, Dziema H, Collamore M, Burgoon PW, Obrietan K (2002). The p42/44 mitogen-activated protein kinase pathway couples photic input to circadian clock entrainment. J Biol Chem.

[CR53] Cade JFJ (1949). Lithium salts in the treatment of psychotic excitement. Med J Aust.

[CR54] Cadet JL (2016). Epigenetics of stress, addiction, and resilience: therapeutic implications. Mol Neurobiol.

[CR55] Caldji C, Tannenbaum B, Sharma S, Francis D, Plotsky PM, Meaney MJ (1998). Maternal care during infancy regulates the development of neural systems mediating the expression of fearfulness in the rat. Proc Natl Acad Sci.

[CR56] Caldji C, Diorio J, Meaney MJ (2000). Variations in maternal care in infancy regulate the development of stress reactivity. Biol Psychiatry.

[CR57] Caldji C, Francis D, Sharma S, Plotsky PM, Meaney MJ (2000). The effects of early rearing environment on the development of GABAA and central benzodiazepine receptor levels and novelty-induced fearfulness in the rat. Neuropsychopharmacol.

[CR58] Campani D, Esposito I, Boggi U, Cecchetti D, Menicagli M, De Negri F (2001). Bcl-2 expression in pancreas development and pancreatic cancer progression. J Pathol.

[CR59] Cassidy F, Murry E, Forest K, Carroll BJ (1998). Signs and symptoms of mania in pure and mixed episodes. J Affect Disord.

[CR60] Challet E (2007). Minireview: entrainment of the suprachiasmatic clockwork in diurnal and nocturnal mammals. Endocrinology.

[CR61] Chen G, Zeng WZ, Yuan PX, Huang LD, Jiang YM, Zhao ZH (1999). The mood-stabilizing agents lithium and valproate robustly increase the levels of the neuroprotective protein bcl-2 in the CNS. J Neurochem.

[CR62] Cheng C-H, Chan PY, Liu CY, Hsu SC (2016). Auditory sensory gating in patients with bipolar disorders: a meta-analysis. J Affect Disord.

[CR63] Cheniaux E, Filgueiras A, da Silva RD, Silveira LAS, Nunes ALS, Landeira-Fernandez J (2014). Increased energy/activity, not mood changes, is the core feature of mania. J Affect Disord.

[CR64] Christie JE, Whalley LJ, Hunter R, Bennie J, Fink G (1989). Sulpiride treatment of acute mania with a comparison of the effects on plasma hormone concentrations of lithium and sulpiride treatment. J Affect Disord.

[CR65] Cipriani A, Barbui C, Salanti G, Rendell J, Brown R, Stockton S (2011). Comparative efficacy and acceptability of antimanic drugs in acute mania: a multiple-treatments meta-analysis. Lancet Lond Engl.

[CR66] Claassen DO, van den Wildenberg WPM, Ridderinkhof KR, Jessup CK, Harrison MB, Wooten GF (2011). The risky business of dopamine agonists in Parkinson disease and impulse control disorders. Behav Neurosci.

[CR67] Clarke AS, Schneider ML (1993). Prenatal stress has long-term effects on behavioral responses to stress in juvenile rhesus monkeys. Dev Psychobiol.

[CR68] Coe CL, Kramer M, Czéh B, Gould E, Reeves AJ, Kirschbaum C (2003). Prenatal stress diminishes neurogenesis in the dentate gyrus of juvenile rhesus monkeys. Biol Psychiatry.

[CR69] Cohen NJ, Lojkasek M, Zadeh ZY, Pugliese M, Kiefer H (2008). Children adopted from China: a prospective study of their growth and development. J Child Psychol Psychiatry.

[CR70] Colombo C, Benedetti F, Barbini B, Campori E, Smeraldi E (1999). Rate of switch from depression into mania after therapeutic sleep deprivation in bipolar depression. Psychiatry Res.

[CR71] Coogan AN, Piggins HD (2004). MAP kinases in the mammalian circadian system–key regulators of clock function. J Neurochem.

[CR72] Cooper S, Robison AJ, Mazei-Robison MS (2017). Reward circuitry in addiction.

[CR73] Cope ZA, Powell SB, Young JW (2016). Modeling neurodevelopmental cognitive deficits in tasks with cross-species translational validity. Genes Brain Behav.

[CR74] Coque L, Mukherjee S, Cao J-L, Spencer S, Marvin M, Falcon E (2011). Specific role of VTA dopamine neuronal firing rates and morphology in the reversal of anxiety-related, but not depression-related behavior in the clockΔ19 mouse model of mania. Neuropsychopharmacology.

[CR75] Corp SA, Gitlin MJ, Altshuler LL (2014). A review of the use of stimulants and stimulant alternatives in treating bipolar depression and major depressive disorder. J Clin Psychiatry.

[CR76] Cosgrove VE, Kelsoe JR, Suppes T (2016). Toward a valid animal model of bipolar disorder: how the research domain criteria help bridge the clinical-basic science divide. Biol Psychiatry.

[CR77] Costello CG (1982). Social factors associated with depression: a retrospective community study. Psychol Med.

[CR78] Cotella EM, Mestres Lascano I, Franchioni L, Levin GM, Suárez MM (2013). Long-term effects of maternal separation on chronic stress response suppressed by amitriptyline treatment. Stress Amst Neth.

[CR79] Cotter D, Takei N, Farrell M, Sham P, Quinn P, Larkin C (1995). Does prenatal exposure to influenza in mice induce pyramidal cell disarray in the dorsal hippocampus?. Schizophr Res.

[CR80] Cousins DA, Butts K, Young AH (2009). The role of dopamine in bipolar disorder. Bipolar Disord.

[CR81] Crawford LK, Rahman SF, Beck SG (2013). Social stress alters inhibitory synaptic input to distinct subpopulations of raphe serotonin neurons. ACS Chem Neurosci.

[CR82] Crump C, Sundquist K, Winkleby MA, Sundquist J (2013). Comorbidities and mortality in bipolar disorder: a Swedish national cohort study. JAMA Psychiatry.

[CR83] D’Sa C, Duman RS (2002). Antidepressants and neuroplasticity. Bipolar Disord.

[CR84] Davies C, Sanger DJ, Steinberg H, Tomkiewicz M, U’Prichard DC (1974). Lithium and alpha-methyl-p-tyrosine prevent “manic” activity in rodents. Psychopharmacologia.

[CR85] De Bellis MD, Baum AS, Birmaher B, Keshavan MS, Eccard CH, Boring AM (1999). Developmental traumatology part I: biological stress systems. Biol Psychiatry.

[CR86] De Bellis MD, Keshavan MS, Clark DB, Casey BJ, Giedd JN, Boring AM (1999). A.E. bennett research award. Developmental traumatology. Part II: brain development. Biol Psychiatry.

[CR87] De Bellis MD, Hooper SR, Spratt EG, Woolley DP (2009). Neuropsychological findings in childhood neglect and their relationships to pediatric PTSD. J Int Neuropsychol Soc.

[CR88] de Fernández Cossío L, Guzmán A, van der Veldt S, Luheshi GN (2017). Prenatal infection leads to ASD-like behavior and altered synaptic pruning in the mouse offspring. Brain Behav Immun.

[CR89] DeVries AC, Joh HD, Bernard O, Hattori K, Hurn PD, Traystman RJ (2001). Social stress exacerbates stroke outcome by suppressing Bcl-2 expression. Proc Natl Acad Sci USA.

[CR90] Di Chiara G, Loddo P, Tanda G (1999). Reciprocal changes in prefrontal and limbic dopamine responsiveness to aversive and rewarding stimuli after chronic mild stress: implications for the psychobiology of depression. Biol Psychiatry.

[CR91] Dickinson T, Becerra R, Coombes J (2017). Executive functioning deficits among adults with bipolar disorder (types I and II): a systematic review and meta-analysis. J Affect Disord.

[CR92] Diz-Chaves Y, Pernía O, Carrero P, Garcia-Segura LM (2012). Prenatal stress causes alterations in the morphology of microglia and the inflammatory response of the hippocampus of adult female mice. J Neuroinflamm.

[CR93] do Couto FS, Batalha VL, Valadas JS, Data-Franca J, Ribeiro JA, Lopes LV (2012). Escitalopram improves memory deficits induced by maternal separation in the rat. Eur J Pharmacol.

[CR94] Doble BW, Woodgett JR (2003). GSK-3: tricks of the trade for a multi-tasking kinase. J Cell Sci.

[CR95] Du J, Gray NA, Falke CA, Chen W, Yuan P, Szabo ST (2004). Modulation of synaptic plasticity by antimanic agents: the role of AMPA glutamate receptor subunit 1 synaptic expression. J Neurosci Off J Soc Neurosci.

[CR96] Dulcis D, Jamshidi P, Leutgeb S, Spitzer NC (2013). Neurotransmitter switching in the adult brain regulates behavior. Science.

[CR97] Duman RS, Monteggia LM (2006). A neurotrophic model for stress-related mood disorders. Biol Psychiatry.

[CR98] Duman RS, Aghajanian GK, Sanacora G, Krystal JH (2016). Synaptic plasticity and depression: new insights from stress and rapid-acting antidepressants. Nat Med.

[CR99] Dumbell R, Matveeva O, Oster H (2016). Circadian clocks, stress, and immunity. Front Endocrinol.

[CR100] Eiland L, Romeo RD (2013). Stress and the developing adolescent brain. Neuroscience.

[CR101] Einat H (2014). New ways of modeling bipolar disorder. Harv Rev Psychiatry.

[CR102] Einat H, Manji HK (2006). Cellular plasticity cascades: genes-to-behavior pathways in animal models of bipolar disorder. Biol Psychiatry.

[CR103] Einat H, Yuan P, Manji HK (2005). Increased anxiety-like behaviors and mitochondrial dysfunction in mice with targeted mutation of the Bcl-2 gene: further support for the involvement of mitochondrial function in anxiety disorders. Behav Brain Res.

[CR104] Einat H, Kronfeld-Schor N, Eilam D (2006). Sand rats see the light: short photoperiod induces a depression-like response in a diurnal rodent. Behav Brain Res.

[CR105] El Khoury A, Gruber SHM, Mørk A, Mathé AA (2006). Adult life behavioral consequences of early maternal separation are alleviated by escitalopram treatment in a rat model of depression. Prog Neuropsychopharmacol Biol Psychiatry.

[CR106] Engel SR, Creson TK, Hao Y, Shen Y, Maeng S, Nekrasova T (2008). The extracellular signal-regulated kinase pathway contributes to the control of behavioral excitement. Mol Psychiatry.

[CR107] Eßlinger M, Wachholz S, Manitz M-P, Plümper J, Sommer R, Juckel G (2016). Schizophrenia associated sensory gating deficits develop after adolescent microglia activation. Brain Behav Immun.

[CR108] Etain B, Milhiet V, Bellivier F, Leboyer M (2011). Genetics of circadian rhythms and mood spectrum disorders. Eur Neuropsychopharmacol.

[CR109] Fatima M, Srivastav S, Mondal AC (2017). Prenatal stress and depression associated neuronal development in neonates. Int J Dev Neurosci.

[CR110] Ferguson SM, Brodkin JD, Lloyd GK, Menzaghi F (2000). Antidepressant-like effects of the subtype-selective nicotinic acetylcholine receptor agonist, SIB-1508Y, in the learned helplessness rat model of depression. Psychopharmacology.

[CR111] Fisher G, Pelonero AL, Ferguson C (1991). Mania precipitated by prednisone and bromocriptine. Gen Hosp Psychiatry.

[CR112] Flemenbaum A (1974). Does lithium block the effects of amphetamine? A report of three cases. Am J Psychiatry.

[CR113] Floresco SB, Phillips AG (2001). Delay-dependent modulation of memory retrieval by infusion of a dopamine D1 agonist into the rat medial prefrontal cortex. Behav Neurosci.

[CR114] Francis DD, Meaney MJ (1999). Maternal care and the development of stress responses. Curr Opin Neurobiol.

[CR115] Frank E, Swartz HA, Kupfer DJ (2000). Interpersonal and social rhythm therapy: managing the chaos of bipolar disorder. Biol Psychiatry.

[CR116] Frank E, Swartz HA, Boland E (2007). Interpersonal and social rhythm therapy: an intervention addressing rhythm dysregulation in bipolar disorder. Dialogues Clin Neurosci.

[CR117] Freund N, Thompson BS, Sonntag K, Meda S, Andersen SL (2016). When the party is over: depressive-like states in rats following termination of cortical D1 receptor overexpression. Psychopharmacology.

[CR118] Frey BN, Valvassori SS, Réus GZ, Martins MR, Petronilho FC, Bardini K (2006). Effects of lithium and valproate on amphetamine-induced oxidative stress generation in an animal model of mania. J Psychiatry Neurosci JPN.

[CR119] Frey BN, Andreazza AC, Houenou J, Jamain S, Goldstein BI, Frye MA (2013). Biomarkers in bipolar disorder: a positional paper from the international society for bipolar disorders biomarkers task force. Aust NZ J Psychiatry.

[CR120] Fries GR, Valvassori SS, Bock H, Stertz L, da Silva Magalhães PV, Mariot E (2015). Memory and brain-derived neurotrophic factor after subchronic or chronic amphetamine treatment in an animal model of mania. J Psychiatr Res.

[CR121] Frye CA, Wawrzycki J (2003). Effect of prenatal stress and gonadal hormone condition on depressive behaviors of female and male rats. Horm Behav.

[CR122] Fuller Torrey E, Rawlings RR, Ennis JM, Merrill DD, Flores DS (1996). Birth seasonality in bipolar disorder, schizophrenia, schizoaffective disorder and stillbirths. Schizophr Res.

[CR123] Gainetdinov RR, Mohn AR, Caron MG (2001). Genetic animal models: focus on schizophrenia. Trends Neurosci.

[CR124] Gerner RH, Post RM, Bunney WE (1976). A dopaminergic mechanism in mania. Am J Psychiatry.

[CR125] Gessa GL, Pani L, Fadda P, Fratta W (1995). Sleep deprivation in the rat: an animal model of mania. Eur Neuropsychopharmacol.

[CR126] Gilbert R, Widom CS, Browne K, Fergusson D, Webb E, Janson S (2009). Burden and consequences of child maltreatment in high-income countries. Lancet.

[CR127] Giros B, Jaber M, Jones SR, Wightman RM, Caron MG (1996). Hyperlocomotion and indifference to cocaine and amphetamine in mice lacking the dopamine transporter. Nature.

[CR128] Goetze U, Tölle R (1987). Circadian rhythm of free urinary cortisol, temperature and heart rate in endogenous depressives and under antidepressant therapy. Neuropsychobiology.

[CR129] Goldberg JF, Burdick KE, Endick CJ (2004). Preliminary randomized, double-blind, placebo-controlled trial of pramipexole added to mood stabilizers for treatment-resistant bipolar depression. Am J Psychiatry.

[CR130] Goldman-Rakic PS (1999). The “psychic” neuron of the cerebral cortex. Ann NY Acad Sci.

[CR131] Goldstein BI, Strober MA, Birmaher B, Axelson DA, Esposito-Smythers C, Goldstein TR (2008). Substance use disorders among adolescents with bipolar spectrum disorders. Bipolar Disord.

[CR132] Goldstein BI, Kemp DE, Soczynska JK, McIntyre RS (2009). Inflammation and the phenomenology, pathophysiology, comorbidity, and treatment of bipolar disorder: a systematic review of the literature. J Clin Psychiatry.

[CR133] Gonul AS, Coburn K, Kula M (2009). Cerebral blood flow, metabolic, receptor, and transporter changes in bipolar disorder: the role of PET and SPECT studies. Int Rev Psychiatry Abingdon Engl.

[CR134] Gonzalez R (2014). The relationship between bipolar disorder and biological rhythms. J Clin Psychiatry.

[CR135] Goodwin GM, Martinez-Aran A, Glahn DC, Vieta E (2008). Cognitive impairment in bipolar disorder: neurodevelopment or neurodegeneration? An ECNP expert meeting report. Eur Neuropsychopharmacol.

[CR136] Gould TD, Einat H (2007). Animal models of bipolar disorder and mood stabilizer efficacy: a critical need for improvement. Neurosci Biobehav Rev.

[CR137] Gould TJ, Keith RA, Bhat RV (2001). Differential sensitivity to lithium’s reversal of amphetamine-induced open-field activity in two inbred strains of mice. Behav Brain Res.

[CR138] Gray NA, Du J, Falke CS, Yuan P, Manji HK (2003). Lithium regulates total and synaptic expression of the AMPA glutamate receptor GluR2 in vitro and in vivo. Ann NY Acad Sci.

[CR139] Green HF, Nolan YM (2012). GSK-3 mediates the release of IL-1β, TNF-α and IL-10 from cortical glia. Neurochem Int.

[CR140] Green JG, McLaughlin KA, Berglund PA, Gruber MJ, Sampson NA, Zaslavsky AM (2010). Childhood adversities and adult psychopathology in the national comorbidity survey replication (NCS-R) I: associations with first onset of DSM-IV disorders. Arch Gen Psychiatry.

[CR141] Grunze H, Vieta E, Goodwin GM, Bowden C, Licht RW, Möller H-J (2013). The world federation of societies of biological psychiatry (WFSBP) guidelines for the biological treatment of bipolar disorders: update 2012 on the long-term treatment of bipolar disorder. World J Biol Psychiatry.

[CR142] Guan L, Jia N, Zhao X, Zhang X, Tang G, Yang L (2013). The involvement of ERK/CREB/Bcl-2 in depression-like behavior in prenatally stressed offspring rats. Brain Res Bull.

[CR143] Gutman DA, Nemeroff CB (2002). Neurobiology of early life stress: rodent studies. Semin Clin Neuropsychiatry.

[CR144] Haarman BC, der Lek Riemersma-Van RF, de Groot JC, Ruhé HG, Klein HC, Zandstra TE (2014). Neuroinflammation in bipolar disorder—A [(11)C]-(R)-PK11195 positron emission tomography study. Brain Behav Immun.

[CR145] Hannestad JO, Cosgrove KP, DellaGioia NF, Perkins E, Bois F, Bhagwagar Z (2013). Changes in the cholinergic system between bipolar depression and euthymia as measured with [123I]5IA single photon emission computed tomography. Biol Psychiatry.

[CR146] Hao LY, Hao XQ, Li SH, Li XH (2010). Prenatal exposure to lipopolysaccharide results in cognitive deficits in age-increasing offspring rats. Neuroscience.

[CR147] Harlow HF, Zimmermann RR (1959). Affectional responses in the infant monkey: orphaned baby monkeys develop a strong and persistent attachment to inanimate surrogate mothers. Science.

[CR148] Harrison EL, Baune BT (2014). Modulation of early stress-induced neurobiological changes: a review of behavioural and pharmacological interventions in animal models. Transl Psychiatry.

[CR149] Harrison PJ, Cipriani A, Harmer CJ, Nobre AC, Saunders K, Goodwin GM (2016). Innovative approaches to bipolar disorder and its treatment. Ann NY Acad Sci.

[CR150] Heim C, Nemeroff CB (2002). Neurobiology of early life stress: clinical studies. Semin Clin Neuropsychiatry.

[CR151] Hicks RA, Moore JD, Hayes C, Phillips N, Hawkins J (1979). REM sleep deprivation increases aggressiveness in male rats. Physiol Behav.

[CR152] Himmerich H, Bartsch S, Hamer H, Mergl R, Schönherr J, Petersein C (2013). Impact of mood stabilizers and antiepileptic drugs on cytokine production in-vitro. J Psychiatr Res.

[CR153] Hoang U, Stewart R, Goldacre MJ (2011). Mortality after hospital discharge for people with schizophrenia or bipolar disorder: retrospective study of linked English hospital episode statistics, 1999–2006. BMJ.

[CR154] Hoertnagl CM, Oberheinricher S, Hofer A (2014). Soziale Kognition bei PatientInnen mit affektiven Störungen: Teil II: Bipolar affektive Störung. Neuropsychiatrie.

[CR155] Hofer MA (1970). Physiological responses of infant rats to separation from their mothers. Science.

[CR156] Hofer MA (1975). Studies on how early maternal separation produces behavioral change in young rats. Psychosom Med.

[CR157] Hofer MA (1976). The organization of sleep and wakefulness after maternal separation in young rats. Dev Psychobiol.

[CR158] Hofer MA (2005). The psychobiology of early attachment. Clin Neurosci Res.

[CR159] Hollis F, Kabbaj M (2014). Social defeat as an animal model for depression. ILAR J.

[CR160] Hollis F, Wang H, Dietz D, Gunjan A, Kabbaj M (2010). The effects of repeated social defeat on long-term depressive-like behavior and short-term histone modifications in the hippocampus in male Sprague-Dawley rats. Psychopharmacology.

[CR161] Holmes MK, Erickson K, Luckenbaugh DA, Drevets WC, Bain EE, Cannon DM (2008). A comparison of cognitive functioning in medicated and unmedicated subjects with bipolar depression. Bipolar Disord.

[CR162] Horger BA, Roth RH (1996). The role of mesoprefrontal dopamine neurons in stress. Crit Rev Neurobiol.

[CR163] Horschitz S, Hummerich R, Lau T, Rietschel M, Schloss P (2005). A dopamine transporter mutation associated with bipolar affective disorder causes inhibition of transporter cell surface expression. Mol Psychiatry.

[CR164] Hovens JGFM, Wiersma JE, Giltay EJ, van Oppen P, Spinhoven P, Penninx BWJH (2010). Childhood life events and childhood trauma in adult patients with depressive, anxiety and comorbid disorders vs. controls. Acta Psychiatr Scand.

[CR165] Huot RL, Thrivikraman KV, Meaney MJ, Plotsky PM (2001). Development of adult ethanol preference and anxiety as a consequence of neonatal maternal separation in long evans rats and reversal with antidepressant treatment. Psychopharmacology.

[CR166] Huot RL, Plotsky PM, Lenox RH, McNamara RK (2002). Neonatal maternal separation reduces hippocampal mossy fiber density in adult Long Evans rats. Brain Res.

[CR167] Husum H, Mathé AA (2002). Early life stress changes concentrations of neuropeptide Y and corticotropin-releasing hormone in adult rat brain. Lithium treatment modifies these changes. Neuropsychopharmacology.

[CR168] Jacobs D, Silverstone T (1986). Dextroamphetamine-induced arousal in human subjects as a model for mania. Psychol Med.

[CR169] Janowsky D, Davis J, El-Yousef MK, Sekerke HJ (1972). A cholinergic–adrenergic hypothesis of mania and depression. Lancet.

[CR170] Janowsky DS, El-Yousef MK, Davis JM (1974). Acetylcholine and depression. Psychosom Med.

[CR171] Janowsky DS, Overstreet DH, Nurnberger JI (1994). Is cholinergic sensitivity a genetic marker for the affective disorders?. Am J Med Genet.

[CR172] Jia N, Li Q, Sun H, Song Q, Tang G, Sun Q (2015). Alterations of group I mGluRs and BDNF associated with behavioral abnormity in prenatally stressed offspring rats. Neurochem Res.

[CR173] Joyce PR, Fergusson DM, Woollard G, Abbott RM, Horwood LJ, Upton J (1995). Urinary catecholamines and plasma hormones predict mood state in rapid cycling bipolar affective disorder. J Affect Disord.

[CR174] Juckel G, Hegerl U, Mavrogiorgou P, Gallinat J, Mager T, Tigges P (2000). Clinical and biological findings in a case with 48-hour bipolar ultrarapid cycling before and during valproate treatment. J Clin Psychiatry.

[CR175] Kaffman A, Meaney MJ (2007). Neurodevelopmental sequelae of postnatal maternal care in rodents: clinical and research implications of molecular insights. J Child Psychol Psychiatry.

[CR176] Kaiser T, Feng G (2015). Modeling psychiatric disorders for developing effective treatments. Nat Med.

[CR177] Kalinichev M, Easterling KW, Plotsky PM, Holtzman SG (2002). Long-lasting changes in stress-induced corticosterone response and anxiety-like behaviors as a consequence of neonatal maternal separation in Long-Evans rats. Pharmacol Biochem Behav.

[CR178] Kato T, Kubota M, Kasahara T (2007). Animal models of bipolar disorder. Neurosci Biobehav Rev.

[CR179] Kaufman J, Charney D (2001). Effects of early stress on brain structure and function: implications for understanding the relationship between child maltreatment and depression. Dev Psychopathol.

[CR180] Kendler KS, Karkowski LM, Prescott CA (1999). Causal relationship between stressful life events and the onset of major depression. Am J Psychiatry.

[CR181] Kernie SG, Liebl DJ, Parada LF (2000). BDNF regulates eating behavior and locomotor activity in mice. EMBO J.

[CR182] Kessler RC, Crum RM, Warner LA, Nelson CB, Schulenberg J, Anthony JC (1997). Lifetime co-occurrence of DSM-III-R alcohol abuse and dependence with other psychiatric disorders in the national comorbidity survey. Arch Gen Psychiatry.

[CR183] Kilbey MM, Ellinwood EH (1977). Reverse tolerance to stimulant-induced abnormal behavior. Life Sci.

[CR184] King DP, Zhao Y, Sangoram AM, Wilsbacher LD, Tanaka M, Antoch MP (1997). Positional cloning of the mouse circadian clock gene. Cell.

[CR185] Kittel-Schneider S, Schreck S, Ziegler C, Weißflog L, Hilscher M, Schwarz R (2015). Lithium-induced clock gene expression in lymphoblastoid cells of bipolar affective patients. Pharmacopsychiatry.

[CR186] Klein PS, Melton DA (1996). A molecular mechanism for the effect of lithium on development. Proc Natl Acad Sci.

[CR187] Klemfuss H (1992). Rhythms and the pharmacology of lithium. Pharmacol Ther.

[CR188] Kneeland RE, Fatemi SH (2013). Viral infection, inflammation and schizophrenia. Prog Neuropsychopharmacol Biol Psychiatry.

[CR189] Koehl M, Darnaudéry M, Dulluc J, Van R, Moal ML, Maccari S (1999). Prenatal stress alters circadian activity of hypothalamo-pituitary- adrenal axis and hippocampal corticosteroid receptors in adult rats of both gender. J Neurobiol.

[CR190] Kohl S, Heekeren K, Klosterkötter J, Kuhn J (2013). Prepulse inhibition in psychiatric disorders—apart from schizophrenia. J Psychiatr Res.

[CR191] Kozikowski AP, Gaisina IN, Yuan H, Petukhov PA, Blond SY, Fedolak A (2007). Structure-based design leads to the identification of lithium mimetics that block mania-like effects in rodents. possible new GSK-3beta therapies for bipolar disorders. J Am Chem Soc.

[CR200] Kraepelin E. Psychiatrie. ein Lehrbuch für Studierende und Ärzte/von Emil Kraepelin. Leipzig Barth; 1909. http://widgets.ebscohost.com/prod/customerspecific/s9118275/vpn.php?url=http://search.ebscohost.com/login.aspx?direct=true&db=cat04752a&AN=rub.1372991&lang=de&site=eds-live.

[CR192] Kripke DF, Mullaney DJ, Atkinson M, Wolf S (1978). Circadian rhythm disorders in manic-depressives. Biol Psychiatry.

[CR193] Kripke DF, Nievergelt CM, Joo E, Shekhtman T, Kelsoe JR (2009). Circadian polymorphisms associated with affective disorders. J Circadian Rhythm.

[CR194] Kuhn CM, Pauk J, Schanberg SM (1990). Endocrine responses to mother-infant separation in developing rats. Dev Psychobiol.

[CR195] Kupfer DJ (2005). The increasing medical burden in bipolar disorder. JAMA.

[CR196] Ladd CO, Huot RL, Thrivikraman KV, Nemeroff CB, Meaney MJ, Plotsky PM (2000). Long-term behavioral and neuroendocrine adaptations to adverse early experience. Prog Brain Res.

[CR197] Ladd CO, Huot RL, Thrivikraman KV, Nemeroff CB, Plotsky PM (2004). Long-term adaptations in glucocorticoid receptor and mineralocorticoid receptor mRNA and negative feedback on the hypothalamo-pituitary-adrenal axis following neonatal maternal separation. Biol Psychiatry.

[CR198] Leach G, Adidharma W, Yan L (2013). Depression-like responses induced by daytime light deficiency in the diurnal grass rat (*Arvicanthis niloticus*). PLoS ONE.

[CR199] Lee AL, Ogle WO, Sapolsky RM (2002). Stress and depression: possible links to neuron death in the hippocampus. Bipolar Disord.

[CR201] Lemaire V, Koehl M, Moal ML, Abrous DN (2000). Prenatal stress produces learning deficits associated with an inhibition of neurogenesis in the hippocampus. Proc Natl Acad Sci.

[CR202] Le-Niculescu H, McFarland MJ, Ogden CA, Balaraman Y, Patel S, Tan J (2008). Phenomic, convergent functional genomic, and biomarker studies in a stress-reactive genetic animal model of bipolar disorder and co-morbid alcoholism. Am J Med Genet Part B Neuropsychiatr Genet.

[CR203] Leo D, Gainetdinov RR (2013). Transgenic mouse models for ADHD. Cell Tissue Res.

[CR204] Leuner B, Fredericks PJ, Nealer C, Albin-Brooks C (2014). Chronic gestational stress leads to depressive-like behavior and compromises medial prefrontal cortex structure and function during the postpartum period. PLoS ONE.

[CR205] Leverich GS, McElroy SL, Suppes T, Keck PE, Denicoff KD, Nolen WA (2002). Early physical and sexual abuse associated with an adverse course of bipolar illness. Biol Psychiatry.

[CR206] Lévy F, Melo AI, Galef BG, Madden M, Fleming AS (2003). Complete maternal deprivation affects social, but not spatial, learning in adult rats. Dev Psychobiol.

[CR207] Lien R, Flaisher-Grinberg S, Cleary C, Hejny M, Einat H (2008). Behavioral effects of Bcl-2 deficiency: implications for affective disorders. Pharmacol Rep.

[CR208] Lin Y-L, Wang S (2014). Prenatal lipopolysaccharide exposure increases depression-like behaviors and reduces hippocampal neurogenesis in adult rats. Behav Brain Res.

[CR209] Lin Y-L, Lin S-Y, Wang S (2012). Prenatal lipopolysaccharide exposure increases anxiety-like behaviors and enhances stress-induced corticosterone responses in adult rats. Brain Behav Immun.

[CR210] Lippmann M, Bress A, Nemeroff CB, Plotsky PM, Monteggia LM (2007). Long-term behavioural and molecular alterations associated with maternal separation in rats. Eur J Neurosci.

[CR211] Liu D, Diorio J, Tannenbaum B, Caldji C, Francis D, Freedman A (1997). Maternal care, hippocampal glucocorticoid receptors, and hypothalamic-pituitary-adrenal responses to stress. Science.

[CR212] Liu D, Diorio J, Day JC, Francis DD, Meaney MJ (2000). Maternal care, hippocampal synaptogenesis and cognitive development in rats. Nat Neurosci.

[CR213] Logan RW, McClung CA (2016). Animal models of bipolar mania: the past, present and future. Neuroscience.

[CR214] Logan RW, Williams WP, McClung CA (2014). Circadian rhythms and addiction: mechanistic insights and future directions. Behav Neurosci.

[CR215] Loman MM, Wiik KL, Frenn KA, Pollak SD, Gunnar MR (2009). Postinstitutionalized children’s development: growth, cognitive, and language outcomes. J Dev Behav Pediatr.

[CR216] Loos M, Pattij T, Janssen MCW, Counotte DS, Schoffelmeer ANM, Smit AB (2010). Dopamine receptor D1/D5 gene expression in the medial prefrontal cortex predicts impulsive choice in rats. Cereb Cortex.

[CR217] Lupien SJ, McEwen BS, Gunnar MR, Heim C (2009). Effects of stress throughout the lifespan on the brain, behaviour and cognition. Nat Rev Neurosci.

[CR218] Lyons WE, Mamounas LA, Ricaurte GA, Coppola V, Reid SW, Bora SH (1999). Brain-derived neurotrophic factor-deficient mice develop aggressiveness and hyperphagia in conjunction with brain serotonergic abnormalities. Proc Natl Acad Sci.

[CR219] Macêdo DS, Medeiros CD, Cordeiro RC, Sousa FC, Santos JV, Morais TA (2012). Effects of alpha-lipoic acid in an animal model of mania induced by d-amphetamine. Bipolar Disord.

[CR220] Macêdo DS, de Lucena DF, Queiroz AIG, Cordeiro RC, Araújo MM, Sousa FC (2013). Effects of lithium on oxidative stress and behavioral alterations induced by lisdexamfetamine dimesylate: relevance as an animal model of mania. Prog Neuropsychopharmacol Biol Psychiatry.

[CR221] MacQueen GM, Ramakrishnan K, Ratnasingan R, Chen B, Young LT (2003). Desipramine treatment reduces the long-term behavioural and neurochemical sequelae of early-life maternal separation. Int J Neuropsychopharmacol.

[CR222] Maes M, Mihaylova I, Kubera M, Ringel K (2012). Activation of cell-mediated immunity in depression: association with inflammation, melancholia, clinical staging and the fatigue and somatic symptom cluster of depression. Prog Neuropsychopharmacol Biol Psychiatry.

[CR223] Magariños AM, Li CJ, Gal Toth J, Bath KG, Jing D, Lee FS (2011). Effect of brain-derived neurotrophic factor haploinsufficiency on stress-induced remodeling of hippocampal neurons. Hippocampus.

[CR224] Maier SF, Seligman ME (1976). Learned helplessness: theory and evidence. J Exp Psychol Gen.

[CR225] Malkesman O, Austin DR, Chen G, Manji HK (2009). Reverse translational strategies for developing animal models of bipolar disorder. Dis Model Mech.

[CR226] Malkoff-Schwartz S, Frank E, Anderson B, Sherrill JT, Siegel L, Patterson D (1998). Stressful life events and social rhythm disruption in the onset of manic and depressive bipolar episodes: a preliminary investigation. Arch Gen Psychiatry.

[CR227] Maniglio R (2009). The impact of child sexual abuse on health: a systematic review of reviews. Clin Psychol Rev.

[CR228] Manji HK, Moore GJ, Chen G (2000). Lithium up-regulates the cytoprotective protein Bcl-2 in the CNS in vivo: a role for neurotrophic and neuroprotective effects in manic depressive illness. J Clin Psychiatry.

[CR229] Manji HK, Drevets WC, Charney DS (2001). The cellular neurobiology of depression. Nat Med.

[CR230] Marangoni C, Hernandez M, Faedda GL (2016). The role of environmental exposures as risk factors for bipolar disorder: a systematic review of longitudinal studies. J Affect Disord.

[CR231] Markou A, Koob GF (1991). Postcocaine anhedonia. An animal model of cocaine withdrawal. Neuropsychopharmacology.

[CR232] Marszalek-Grabska M, Gibula-Bruzda E, Jenda M, Gawel K, Kotlinska JH (2016). Memantine improves memory impairment and depressive-like behavior induced by amphetamine withdrawal in rats. Brain Res.

[CR233] Martinek S, Inonog S, Manoukian AS, Young MW (2001). A role for the segment polarity gene shaggy/GSK-3 in the drosophila circadian clock. Cell.

[CR234] Martínez-Arán A, Vieta E, Reinares M, Colom F, Torrent C, Sánchez-Moreno J (2004). Cognitive function across manic or hypomanic, depressed, and euthymic states in bipolar disorder. Am J Psychiatry.

[CR235] Mason WA, Berkson G (1975). Effects of maternal mobility on the development of rocking and other behaviors in rhesus monkeys: a study with artificial mothers. Dev Psychobiol.

[CR236] Masri B, Salahpour A, Didriksen M, Ghisi V, Beaulieu J-M, Gainetdinov RR (2008). Antagonism of dopamine D2 receptor/β-arrestin 2 interaction is a common property of clinically effective antipsychotics. Proc Natl Acad Sci.

[CR237] McCarthy MJ, Welsh DK (2012). Cellular circadian clocks in mood disorders. J Biol Rhythms.

[CR238] McClung CA (2007). Circadian genes, rhythms and the biology of mood disorders. Pharmacol Ther.

[CR239] McClung CA (2013). How might circadian rhythms control mood? Let me count the ways. Biol Psychiatry.

[CR240] McClung CA, Sidiropoulou K, Vitaterna M, Takahashi JS, White FJ, Cooper DC (2005). Regulation of dopaminergic transmission and cocaine reward by the Clock gene. Proc Natl Acad Sci USA.

[CR241] McIntosh J, Anisman H, Merali Z (1999). Short- and long-periods of neonatal maternal separation differentially affect anxiety and feeding in adult rats: gender-dependent effects. Dev Brain Res.

[CR242] McLaughlin KA, Green JG, Gruber MJ, Sampson NA, Zaslavsky AM, Kessler RC (2010). Childhood adversities and adult psychiatric disorders in the national comorbidity survey replication II: associations with persistence of DSM-IV disorders. Arch Gen Psychiatry.

[CR243] Meaney MJ (2001). Maternal care, gene expression, and the transmission of individual differences in stress reactivity across generations. Annu Rev Neurosci.

[CR244] Meaney MJ, Viau V, Bhatnagar S, Betito K, Iny LJ, O’Donnell D (1991). Cellular mechanisms underlying the development and expression of individual differences in the hypothalamic-pituitary-adrenal stress response. J Steroid Biochem Mol Biol.

[CR245] Meerlo P, Overkamp GJF, Daan S, Van Den Hoofdakker RH, Koolhaas JM (1996). Changes in behaviour and body weight following a single or double social defeat in rats. Stress Amst Neth.

[CR246] Meesters Y, Gordijn MC (2016). Seasonal affective disorder, winter type: current insights and treatment options. Psychol Res Behav Manag.

[CR247] Merikangas KR, Akiskal HS, Angst J, Greenberg PE, Hirschfeld RMA, Petukhova M (2007). Lifetime and 12-month prevalence of bipolar spectrum disorder in the national comorbidity survey replication. Arch Gen Psychiatry.

[CR248] Merikangas KR, Jin R, He J-P, Kessler RC, Lee S, Sampson NA (2011). Prevalence and correlates of bipolar spectrum disorder in the world mental health survey initiative. Arch Gen Psychiatry.

[CR249] Messer T, Lammers G, Müller-Siecheneder F, Schmidt R-F, Latifi S (2017). Substance abuse in patients with bipolar disorder: a systematic review and meta-analysis. Psychiatry Res.

[CR250] Meyer U (2014). Prenatal Poly(I:C) exposure and other developmental immune activation models in rodent systems. Biol Psychiatry.

[CR251] Meyer U, Feldon J, Schedlowski M, Yee BK (2005). Towards an immuno-precipitated neurodevelopmental animal model of schizophrenia. Neurosci Biobehav Rev.

[CR252] Miklowitz DJ, Otto MW, Frank E, Reilly-Harrington NA, Wisniewski SR, Kogan JN (2007). Psychosocial treatments for bipolar depression: a 1-year randomized trial from the systematic treatment enhancement program. Arch Gen Psychiatry.

[CR253] Minassian A, Henry BL, Young JW, Masten V, Geyer MA, Perry W (2011). Repeated assessment of exploration and novelty seeking in the human behavioral pattern monitor in bipolar disorder patients and healthy individuals. PLoS ONE.

[CR254] Mineur YS, Obayemi A, Wigestrand MB, Fote GM, Calarco CA, Li AM (2013). Cholinergic signaling in the hippocampus regulates social stress resilience and anxiety- and depression-like behavior. Proc Natl Acad Sci USA.

[CR255] Modabbernia A, Taslimi S, Brietzke E, Ashrafi M (2013). Cytokine alterations in bipolar disorder: a meta-analysis of 30 studies. Biol Psychiatry.

[CR256] Moonat S, Pandey SC (2012). Stress, epigenetics, and alcoholism. Alcohol Res Curr Rev.

[CR257] Morden B, Mullins R, Levine S, Cohen H, Dement W (1968). Effect of REM deprivation on the mating behavior of male rats. Psychophysiology.

[CR258] Mukherjee S, Coque L, Cao J-L, Kumar J, Chakravarty S, Asaithamby A (2010). Knockdown of Clock in the ventral tegmental area through RNA interference results in a mixed state of mania and depression-like behavior. Biol Psychiatry.

[CR259] Mullen PE, Martin JL, Anderson JC, Romans SE, Herbison GP (1996). The long-term impact of the physical, emotional, and sexual abuse of children: a community study. Child Abuse Negl.

[CR260] Mutschler NH, Miczek KA (1998). Withdrawal from a self-administered or non-contingent cocaine binge: differences in ultrasonic distress vocalizations in rats. Psychopharmacology.

[CR261] Nelson CA, Zeanah CH, Fox NA, Marshall PJ, Smyke AT, Guthrie D (2007). Cognitive recovery in socially deprived young children: the Bucharest early intervention project. Science.

[CR262] Nemeroff CB, Heim CM, Thase ME, Klein DN, Rush AJ, Schatzberg AF (2003). Differential responses to psychotherapy versus pharmacotherapy in patients with chronic forms of major depression and childhood trauma. Proc Natl Acad Sci USA.

[CR263] Nestler EJ, Hyman SE (2010). Animal models of neuropsychiatric disorders. Nat Neurosci.

[CR264] Nibuya M, Morinobu S, Duman RS (1995). Regulation of BDNF and trkB mRNA in rat brain by chronic electroconvulsive seizure and antidepressant drug treatments. J Neurosci.

[CR265] Nieoullon A, Coquerel A (2003). Dopamine: a key regulator to adapt action, emotion, motivation and cognition. Curr Opin Neurol.

[CR266] Novick DM, Swartz HA, Frank E (2010). Suicide attempts in bipolar I and bipolar II disorder: a review and meta-analysis of the evidence. Bipolar Disord.

[CR267] Nurnberger JI, Gershon ES, Simmons S, Ebert M, Kessler LR, Dibble ED (1982). Behavioral, biochemical and neuroendocrine responses to amphetamine in normal twins and “well-state” bipolar patients. Psychoneuroendocrinology.

[CR268] Nurnberger JI, Jimerson DC, Simmons-Alling S, Tamminga C, Nadi NS, Lawrence D (1983). Behavioral, physiological, and neuroendocrine responses to arecoline in normal twins and “well state” bipolar patients. Psychiatry Res.

[CR269] O’Brien WT, Harper AD, Jové F, Woodgett JR, Maretto S, Piccolo S (2004). Glycogen synthase kinase-3beta haploinsufficiency mimics the behavioral and molecular effects of lithium. J Neurosci.

[CR270] O’Brien WT, Huang J, Buccafusca R, Garskof J, Valvezan AJ, Berry GT (2011). Glycogen synthase kinase-3 is essential for β-arrestin-2 complex formation and lithium-sensitive behaviors in mice. J Clin Invest.

[CR271] Pachet AK, Wisniewski AM (2003). The effects of lithium on cognition: an updated review. Psychopharmacology.

[CR272] Palmisano M, Pandey SC (2017). Epigenetic mechanisms of alcoholism and stress-related disorders. Alcohol Fayettev N.

[CR273] Parboosing R, Bao Y, Shen L, Schaefer CA, Brown AS (2013). Gestational influenza and bipolar disorder in adult offspring. JAMA Psychiatry.

[CR274] Paspalas CD, Wang M, Arnsten AFT (2013). Constellation of HCN channels and cAMP regulating proteins in dendritic spines of the primate prefrontal cortex: potential substrate for working memory deficits in schizophrenia. Cereb Cortex.

[CR275] Patel JP, Frey BN (2015). Disruption in the blood-brain barrier: the missing link between brain and body inflammation in bipolar disorder?. Neural Plast.

[CR276] Paulson PE, Camp DM, Robinson TE (1991). Time course of transient behavioral depression and persistent behavioral sensitization in relation to regional brain monoamine concentrations during amphetamine withdrawal in rats. Psychopharmacology.

[CR277] Paykel ES (1978). Contribution of life events to causation of psychiatric illness. Psychol Med.

[CR278] Pearlson GD, Wong DF, Tune LE, Ross CA, Chase GA, Links JM (1995). In vivo D2 dopamine receptor density in psychotic and nonpsychotic patients with bipolar disorder. Arch Gen Psychiatry.

[CR279] Pechtel P, Pizzagalli DA (2011). Effects of early life stress on cognitive and affective function: an integrated review of human literature. Psychopharmacology.

[CR280] Peet M, Peters S (1995). Drug-induced mania. Drug Saf.

[CR281] Perry W, Minassian A, Feifel D, Braff DL (2001). Sensorimotor gating deficits in bipolar disorder patients with acute psychotic mania. Biol Psychiatry.

[CR282] Perry W, Minassian A, Paulus MP, Young JW, Kincaid MJ, Ferguson EJ (2009). A reverse-translational study of dysfunctional exploration in psychiatric disorders: from mice to men. Arch Gen Psychiatry.

[CR283] Perry W, Minassian A, Henry B, Kincaid M, Young JW, Geyer MA (2010). Quantifying over-activity in bipolar and schizophrenia patients in a human open field paradigm. Psychiatry Res.

[CR284] Persico AM, Schindler CW, Zaczek R, Brannock MT, Uhl GR (1995). Brain transcription factor gene expression, neurotransmitter levels, and novelty response behaviors: alterations during rat amphetamine withdrawal and following chronic injection stress. Synapse.

[CR285] Phillips ML, Kupfer DJ (2013). Bipolar disorder diagnosis: challenges and future directions. Lancet.

[CR286] Pirkola S, Isometsä E, Aro H, Kestilä L, Hämäläinen J, Veijola J (2005). Childhood adversities as risk factors for adult mental disorders: results from the health 2000 study. Soc Psychiatry Psychiatr Epidemiol.

[CR287] Porsolt RD, Le Pichon M, Jalfre M (1977). Depression: a new animal model sensitive to antidepressant treatments. Nature.

[CR288] Post RM (1990). Sensitization and kindling perspectives for the course of affective illness: toward a new treatment with the anticonvulsant carbamazepine. Pharmacopsychiatry.

[CR289] Post RM (1992). Transduction of psychosocial stress into the neurobiology of recurrent affective disorder. Am J Psychiatry.

[CR290] Powell SB, Young JW, Ong JC, Caron MG, Geyer MA (2008). Atypical antipsychotics clozapine and quetiapine attenuate prepulse inhibition deficits in dopamine transporter knockout mice. Behav Pharmacol.

[CR291] Prickaerts J, Moechars D, Cryns K, Lenaerts I, van Craenendonck H, Goris I (2006). Transgenic mice overexpressing glycogen synthase kinase 3β: a putative model of hyperactivity and mania. J Neurosci.

[CR292] Puig MV, Rose J, Schmidt R, Freund N. Dopamine modulation of learning and memory in the prefrontal cortex: insights from studies in primates, rodents, and birds. Front Neural Circuits. 2014;8(93). http://www.frontiersin.org/neural_circuits/10.3389/fncir.2014.00093/abstract. 10.3389/fncir.2014.00093 PMC4122189 25140130

[CR293] Queiroz AIG, de Araújo MM, da Silva Araújo T, de Souza GC, Cavalcante LM, Machado MD (2015). GBR 12909 administration as an animal model of bipolar mania: time course of behavioral, brain oxidative alterations and effect of mood stabilizing drugs. Metab Brain Dis.

[CR294] Raiteri M, Bertollini A, Angelini F, Levi G (1975). d-Amphetamine as a releaser or reuptake inhibitor of biogenic amines in synaptosomes. Eur J Pharmacol.

[CR295] Raja M, Bentivoglio AR (2012). Impulsive and compulsive behaviors during dopamine replacement treatment in Parkinson’s disease and other disorders. Curr Drug Saf.

[CR296] Rajkowska G (2002). Cell pathology in bipolar disorder. Bipolar Disord.

[CR297] Ralph RJ, Paulus MP, Fumagalli F, Caron MG, Geyer MA (2001). Prepulse inhibition deficits and perseverative motor patterns in dopamine transporter knock-out mice: differential effects of D1 and D2 receptor antagonists. J Neurosci.

[CR298] Ralph-Williams RJ, Paulus MP, Zhuang X, Hen R, Geyer MA (2003). Valproate attenuates hyperactive and perseverative behaviors in mutant mice with a dysregulated dopamine system. Biol Psychiatry.

[CR299] Rao JS, Kellom M, Reese EA, Rapoport SI, Kim H-W (2012). Dysregulated glutamate and dopamine transporters in postmortem frontal cortex from bipolar and schizophrenic patients. J Affect Disord.

[CR300] Regier DA, Farmer ME, Rae DS, Locke BZ, Keith SJ, Judd LL (1990). Comorbidity of mental disorders with alcohol and other drug abuse. Results from the epidemiologic catchment area (ECA) study. JAMA.

[CR301] Remus JL, Dantzer R. Inflammation models of depression in rodents: relevance to psychotropic drug discovery. Int J Neuropsychopharmacol. 2016;19(9). https://academic.oup.com/ijnp/article/19/9/pyw028/2488254/Inflammation-Models-of-Depression-in-Rodents. Accessed 23 May 2017. 10.1093/ijnp/pyw028 PMC5043641 27026361

[CR302] Rezin GT, Furlanetto CB, Scaini G, Valvassori SS, Gonçalves CL, Ferreira GK (2014). Fenproporex increases locomotor activity and alters energy metabolism, and mood stabilizers reverse these changes: a proposal for a new animal model of mania. Mol Neurobiol.

[CR303] Ripperger JA, Shearman LP, Reppert SM, Schibler U (2000). CLOCK, an essential pacemaker component, controls expression of the circadian transcription factor DBP. Genes Dev.

[CR304] Romeo RD, Mueller A, Sisti HM, Ogawa S, McEwen BS, Brake WG (2003). Anxiety and fear behaviors in adult male and female C57BL/6 mice are modulated by maternal separation. Horm Behav.

[CR305] Rondi-Reig L, Mariani J (2002). To die or not to die, does it change the function? Behavior of transgenic mice reveals a role for developmental cell death. Brain Res Bull.

[CR306] Rondi-Reig L, Lemaigre Dubreuil Y, Martinou JC, Delhaye-Bouchaud N, Caston J, Mariani J (1997). Fear decrease in transgenic mice overexpressing bcl-2 in neurons. Neuroreport.

[CR307] Roni MA, Rahman S (2014). The effects of lobeline on nicotine withdrawal-induced depression-like behavior in mice. Psychopharmacology.

[CR308] Ronovsky M, Berger S, Molz B, Berger A, Pollak DD (2016). Animal models of maternal immune activation in depression research. Curr Neuropharmacol.

[CR309] Ronovsky M, Berger S, Zambon A, Reisinger SN, Horvath O, Pollak A (2017). Maternal immune activation transgenerationally modulates maternal care and offspring depression-like behavior. Brain Behav Immun.

[CR310] Rose DR, Careaga M, Van de Water J, McAllister K, Bauman MD, Ashwood P (2017). Long-term altered immune responses following fetal priming in a non-human primate model of maternal immune activation. Brain Behav Immun.

[CR311] Rosenthal NE, Sack DA, Gillin JC, Lewy AJ, Goodwin FK, Davenport Y (1984). Seasonal affective disorder. A description of the syndrome and preliminary findings with light therapy. Arch Gen Psychiatry.

[CR312] Rowse AL, Naves R, Cashman KS, McGuire DJ, Mbana T, Raman C (2012). Lithium controls central nervous system autoimmunity through modulation of IFN-γ signaling. PLoS ONE.

[CR313] Roybal K, Theobold D, Graham A, DiNieri JA, Russo SJ, Krishnan V (2007). Mania-like behavior induced by disruption of CLOCK. Proc Natl Acad Sci USA.

[CR314] Ruis MA, te Brake JH, Buwalda B, De Boer SF, Meerlo P, Korte SM (1999). Housing familiar male wildtype rats together reduces the long-term adverse behavioural and physiological effects of social defeat. Psychoneuroendocrinology.

[CR315] Russo-Neustadt AA, Beard RC, Huang YM, Cotman CW (2000). Physical activity and antidepressant treatment potentiate the expression of specific brain-derived neurotrophic factor transcripts in the rat hippocampus. Neuroscience.

[CR316] Rygula R, Szczech E, Kregiel J, Golebiowska J, Kubik J, Popik P (2015). Cognitive judgment bias in the psychostimulant-induced model of mania in rats. Psychopharmacology.

[CR317] Saarelainen T, Hendolin P, Lucas G, Koponen E, Sairanen M, MacDonald E (2003). Activation of the TrkB neurotrophin receptor is induced by antidepressant drugs and is required for antidepressant-induced behavioral effects. J Neurosci.

[CR318] Salloum IM, Cornelius JR, Daley DC, Kirisci L, Himmelhoch JM, Thase ME (2005). Efficacy of valproate maintenance in patients with bipolar disorder and alcoholism: a double-blind placebo-controlled study. Arch Gen Psychiatry.

[CR319] Salvatore P, Tohen M (2007). Khalsa H-MK, Baethge C, Tondo L, Baldessarini RJ. Longitudinal research on bipolar disorders. Epidemiol Psichiatr Soc.

[CR320] Salvi V, Fagiolini A, Swartz HA, Maina G, Frank E (2008). The use of antidepressants in bipolar disorder. J Clin Psychiatry.

[CR321] Sani G, Perugi G, Tondo L (2017). Treatment of bipolar disorder in a lifetime perspective: is lithium still the best choice?. Clin Drug Investig.

[CR322] Savitz J, Solms M, Ramesar R (2005). Neuropsychological dysfunction in bipolar affective disorder: a critical opinion. Bipolar Disord.

[CR323] Schaeffer JC, Cho AK, Fischer JF (1976). Inhibition of synaptosomal accumulation of l-norepinephrine II: N-aryloxyalkylphentermines, quaternary d-amphetamines, and 3-aryloxypropylamines. J Pharm Sci.

[CR324] Schaffer A, Levitt AJ, Boyle M (2003). Influence of season and latitude in a community sample of subjects with bipolar disorder. Can J Psychiatry.

[CR325] Schildkraut JJ (1965). The catecholamine hypothesis of affective disorders: a review of supporting evidence. Am J Psychiatry.

[CR326] Schindler CW, Persico AM, Uhl GR, Goldberg SR (1994). Behavioral assessment of high-dose amphetamine withdrawal: importance of training and testing conditions. Pharmacol Biochem Behav.

[CR327] Schmitt AR, Brewer JD, Bordeaux JS, Baum CL (2014). Staging for cutaneous squamous cell carcinoma as a predictor of sentinel lymph node biopsy results: meta-analysis of american joint committee on cancer criteria and a proposed alternative system. JAMA Dermatol.

[CR328] Schwartz JM, Ksir C, Koob GF, Bloom FE (1982). Changes in locomotor response to beta-endorphin microinfusion during and after opiate abstinence syndrome—a proposal for a model of the onset of mania. Psychiatry Res.

[CR329] Seamans JK, Yang CR (2004). The principal features and mechanisms of dopamine modulation in the prefrontal cortex. Prog Neurobiol.

[CR330] Seiden LS, Sabol KE, Ricaurte GA (1993). Amphetamine: effects on catecholamine systems and behavior. Annu Rev Pharmacol Toxicol.

[CR331] Serretti A, Benedetti F, Mandelli L, Lorenzi C, Pirovano A, Colombo C (2003). Genetic dissection of psychopathological symptoms: insomnia in mood disorders and CLOCK gene polymorphism. Am J Med Genet B Neuropsychiatr Genet.

[CR332] Serretti A, Drago A, De Ronchi D (2009). Lithium pharmacodynamics and pharmacogenetics: focus on inositol mono phosphatase (IMPase), inositol poliphosphatase (IPPase) and glycogen sinthase kinase 3 beta (GSK-3 beta). Curr Med Chem.

[CR333] Shaltiel G, Maeng S, Malkesman O, Pearson B, Schloesser RJ, Tragon T (2008). Evidence for the involvement of the kainate receptor subunit GluR6 (GRIK2) in mediating behavioral displays related to behavioral symptoms of mania. Mol Psychiatry.

[CR334] Sharma AN, Fries GR, Galvez JF, Valvassori SS, Soares JC, Carvalho AF (2016). Modeling mania in preclinical settings: a comprehensive review. Prog Neuropsychopharmacol Biol Psychiatry.

[CR335] Shi L, Fatemi SH, Sidwell RW, Patterson PH (2003). Maternal influenza infection causes marked behavioral and pharmacological changes in the offspring. J Neurosci.

[CR336] Shirayama Y, Chen AC-H, Nakagawa S, Russell DS, Duman RS (2002). Brain-derived neurotrophic factor produces antidepressant effects in behavioral models of depression. J Neurosci.

[CR337] Silverstone T, Fincham J, Wells B, Kyriakides M (1980). The effect of the dopamine receptor blocking drug pimozide on the stimulant and anorectic actions of dextroamphetamine in man. Neuropharmacology.

[CR338] Simhandl C, Radua J, König B, Amann BL (2015). The prevalence and effect of life events in 222 bipolar I and II patients: a prospective, naturalistic 4 year follow-up study. J Affect Disord.

[CR339] Smith MA, Makino S, Kvetnansky R, Post RM (1995). Stress and glucocorticoids affect the expression of brain-derived neurotrophic factor and neurotrophin-3 mRNAs in the hippocampus. J Neurosci.

[CR340] Södersten K, Pålsson E, Ishima T, Funa K, Landén M, Hashimoto K (2014). Abnormality in serum levels of mature brain-derived neurotrophic factor (BDNF) and its precursor proBDNF in mood-stabilized patients with bipolar disorder: a study of two independent cohorts. J Affect Disord.

[CR341] Sonntag KC, Brenhouse HC, Freund N, Thompson BS, Puhl M, Andersen SL (2014). Viral over-expression of D1 dopamine receptors in the prefrontal cortex increase high-risk behaviors in adults: comparison with adolescents. Psychopharmacology.

[CR342] Souetre E, Salvati E, Wehr TA, Sack DA, Krebs B, Darcourt G (1988). Twenty-four-hour profiles of body temperature and plasma TSH in bipolar patients during depression and during remission and in normal control subjects. Am J Psychiatry.

[CR343] Spanagel R, Noori HR, Heilig M (2014). Stress and alcohol interactions: animal studies and clinical significance. Trends Neurosci.

[CR344] Spataro J, Mullen PE, Burgess PM, Wells DL, Moss SA (2004). Impact of child sexual abuse on mental health. Br J Psychiatry.

[CR345] Stahl CE, Redei E, Wang Y, Borlongan CV (2002). Behavioral, hormonal and histological stress markers of anxiety-separation in postnatal rats are reduced by prepro-thyrotropin-releasing hormone 178-199. Neurosci Lett.

[CR346] Stanton ME, Levine S (1990). Inhibition of infant glucocorticoid stress response: specific role of maternal cues. Dev Psychobiol.

[CR347] Stefanski V (2000). Social stress in laboratory rats: hormonal responses and immune cell distribution. Psychoneuroendocrinology.

[CR348] Steingard RJ, Yurgelun-Todd DA, Hennen J, Moore JC, Moore CM, Vakili K (2000). Increased orbitofrontal cortex levels of choline in depressed adolescents as detected by in vivo proton magnetic resonance spectroscopy. Biol Psychiatry.

[CR349] Stertz L, Magalhães PVS, Kapczinski F (2013). Is bipolar disorder an inflammatory condition? The relevance of microglial activation. Curr Opin Psychiatry.

[CR350] Steru L, Chermat R, Thierry B, Simon P (1985). The tail suspension test: a new method for screening antidepressants in mice. Psychopharmacology.

[CR351] Suhara T, Nakayama K, Inoue O, Fukuda H, Shimizu M, Mori A (1992). D1 dopamine receptor binding in mood disorders measured by positron emission tomography. Psychopharmacology.

[CR352] Sulzer D, Sonders MS, Poulsen NW, Galli A (2005). Mechanisms of neurotransmitter release by amphetamines: a review. Prog Neurobiol.

[CR353] Surtees PG, Miller PM, Ingham JG, Kreitman NB, Rennie D, Sashidharan SP (1986). Life events and the onset of affective disorder: a longitudinal general population study. J Affect Disord.

[CR354] Takahashi JS, Hong H-K, Ko CH, McDearmon EL (2008). The genetics of mammalian circadian order and disorder: implications for physiology and disease. Nat Rev Genet.

[CR355] Tanda G, Carboni E, Frau R, Di Chiara G (1994). Increase of extracellular dopamine in the prefrontal cortex: a trait of drugs with antidepressant potential?. Psychopharmacology.

[CR356] Teicher MH (2002). Scars that won’t heal: the neurobiology of child abuse. Sci Am.

[CR357] Thomas GM, Huganir RL (2004). MAPK cascade signalling and synaptic plasticity. Nat Rev Neurosci.

[CR358] Tidey JW, Miczek KA (1997). Acquisition of cocaine self-administration after social stress: role of accumbens dopamine. Psychopharmacology.

[CR359] Tohen M, Goldberg JF, Gonzalez-Pinto Arrillaga AM, Azorin JM, Vieta E, Hardy-Bayle M-C (2003). A 12-week, double-blind comparison of olanzapine vs haloperidol in the treatment of acute mania. Arch Gen Psychiatry.

[CR360] Tornatzky W, Miczek KA (1993). Long-term impairment of autonomic circadian rhythms after brief intermittent social stress. Physiol Behav.

[CR361] Toyoda A (2017). Social defeat models in animal science: what we have learned from rodent models. Anim Sci.

[CR362] Tractenberg SG, Levandowski ML, de Azeredo LA, Orso R, Roithmann LG, Hoffmann ES (2016). An overview of maternal separation effects on behavioural outcomes in mice: evidence from a four-stage methodological systematic review. Neurosci Biobehav Rev.

[CR363] Trejo JL, Cuchillo I, Machín C, Rúa C (2000). Maternal adrenalectomy at the early onset of gestation impairs the postnatal development of the rat hippocampal formation: effects on cell numbers and differentiation, connectivity and calbindin-D28k immunoreactivity. J Neurosci Res.

[CR364] Uno H, Lohmiller L, Thieme C, Kemnitz JW, Engle MJ, Roecker EB (1990). Brain damage induced by prenatal exposure to dexamethasone in fetal rhesus macaques. I. Hippocampus. Dev Brain Res.

[CR365] Valvassori SS, Tonin PT, Varela RB, Carvalho AF, Mariot E, Amboni RT (2015). Lithium modulates the production of peripheral and cerebral cytokines in an animal model of mania induced by dextroamphetamine. Bipolar Disord.

[CR366] van den Dries L, Juffer F, van Ijzendoorn MH, Bakermans-Kranenburg MJ (2010). Infants’ physical and cognitive development after international adoption from foster care or institutions in China. J Dev Behav Pediatr.

[CR367] van Enkhuizen J, Geyer MA, Kooistra K, Young JW (2013). Chronic valproate attenuates some, but not all, facets of mania-like behaviour in mice. Int J Neuropsychopharmacol.

[CR368] van Enkhuizen J, Minassian A, Young JW (2013). Further evidence for ClockΔ19 mice as a model for bipolar disorder mania using cross-species tests of exploration and sensorimotor gating. Behav Brain Res.

[CR369] van Enkhuizen J, Geyer MA, Halberstadt AL, Zhuang X, Young JW (2014). Dopamine depletion attenuates some behavioral abnormalities in a hyperdopaminergic mouse model of bipolar disorder. J Affect Disord.

[CR370] van Enkhuizen J, Henry BL, Minassian A, Perry W, Milienne-Petiot M, Higa KK (2014). Reduced dopamine transporter functioning induces high-reward risk-preference consistent with bipolar disorder. Neuropsychopharmacology.

[CR371] van Enkhuizen J, Geyer MA, Minassian A, Perry W, Henry BL, Young JW (2015). Investigating the underlying mechanisms of aberrant behaviors in bipolar disorder from patients to models: rodent and human studies. Neurosci Biobehav Rev.

[CR372] van Enkhuizen J, Janowsky DS, Olivier B, Minassian A, Perry W, Young JW (2015). The catecholaminergic-cholinergic balance hypothesis of bipolar disorder revisited. Eur J Pharmacol.

[CR373] Van Kammen DP, Murphy DL (1975). Attenuation of the euphoriant and activating effects of d- and l-amphetamine by lithium carbonate treatment. Psychopharmacologia.

[CR374] van Praag HM, Korf J (1975). Central monoamine deficiency in depressions: causative of secondary phenomenon?. Pharmakopsychiatr Neuropsychopharmakol.

[CR375] Vanover KE (1998). Effects of AMPA receptor antagonists on dopamine-mediated behaviors in mice. Psychopharmacology.

[CR376] Varela RB, Valvassori SS, Lopes-Borges J, Fraga DB, Resende WR, Arent CO (2013). Evaluation of acetylcholinesterase in an animal model of mania induced by d-amphetamine. Psychiatry Res.

[CR377] Vetulani J (2013). Early maternal separation: a rodent model of depression and a prevailing human condition. Pharmacol Rep.

[CR378] Vieta E, Ros S, Goikolea JM, Benabarre A, Popova E, Comes M (2005). An open-label study of amisulpride in the treatment of mania. J Clin Psychiatry.

[CR379] Wachholz S, Knorr A, Mengert L, Plümper J, Sommer R, Juckel G (2017). Interleukin-4 is a participant in the regulation of depressive-like behavior. Behav Brain Res.

[CR380] Wakshlak A, Weinstock M (1990). Neonatal handling reverses behavioral abnormalities induced in rats by prenatal stress. Physiol Behav.

[CR381] Wang Y, Huang W, Wang C, Tsai C, Chen C, Chang Y (2009). Inhibiting glycogen synthase kinase-3 reduces endotoxaemic acute renal failure by down-regulating inflammation and renal cell apoptosis. Br J Pharmacol.

[CR382] Wieck A, Grassi-Oliveira R, do Prado CH, Rizzo LB, de Oliveira AS, Kommers-Molina J (2014). Pro-inflammatory cytokines and soluble receptors in response to acute psychosocial stress: differential reactivity in bipolar disorder. Neurosci Lett.

[CR383] Wigger A, Neumann ID (1999). Periodic maternal deprivation induces gender-dependent alterations in behavioral and neuroendocrine responses to emotional stress in adult rats. Physiol Behav.

[CR384] Wirz-Justice A (2006). Biological rhythm disturbances in mood disorders. Int Clin Psychopharmacol.

[CR385] Wise RA, Munn E (1995). Withdrawal from chronic amphetamine elevates baseline intracranial self-stimulation thresholds. Psychopharmacology.

[CR386] Wright JB (1993). Mania following sleep deprivation. Br J Psychiatry J Ment Sci.

[CR387] Wuarin J, Falvey E, Lavery D, Talbot D, Schmidt E, Ossipow V (1992). The role of the transcriptional activator protein DBP in circadian liver gene expression. J Cell Sci Suppl.

[CR388] Wulsin AC, Wick-Carlson D, Packard BA, Morano R, Herman JP (2016). Adolescent chronic stress causes hypothalamo-pituitary-adrenocortical hypo-responsiveness and depression-like behavior in adult female rats. Psychoneuroendocrinology.

[CR389] Yadid G, Overstreet DH, Zangen A (2001). Limbic dopaminergic adaptation to a stressful stimulus in a rat model of depression. Brain Res.

[CR390] Yao J, Pan Y, Ding M, Pang H, Wang B (2013). Meta-analysis shows dopamine receptor D1 gene polymorphism is associated with bipolar disorder but not with schizophrenia. Psychiatry Res.

[CR391] Yin L, Wang J, Klein PS, Lazar MA (2006). Nuclear receptor Rev-erbalpha is a critical lithium-sensitive component of the circadian clock. Science.

[CR392] Young JW, Dulcis D (2015). Investigating the mechanism(s) underlying switching between states in bipolar disorder. Eur J Pharmacol.

[CR393] Young JW, Minassian A, Paulus MP, Geyer MA, Perry W (2007). A reverse-translational approach to bipolar disorder: rodent and human studies in the behavioral pattern monitor. Neurosci Biobehav Rev.

[CR394] Young JW, Goey AKL, Minassian A, Perry W, Paulus MP, Geyer MA (2010). The mania-like exploratory profile in genetic dopamine transporter mouse models is diminished in a familiar environment and reinstated by subthreshold psychostimulant administration. Pharmacol Biochem Behav.

[CR395] Young JW, van Enkhuizen J, Winstanley CA, Geyer MA (2011). Increased risk-taking behavior in dopamine transporter knockdown mice: further support for a mouse model of mania. J Psychopharmacol Oxf Engl.

[CR396] Zarate CA, Payne JL, Singh J, Quiroz JA, Luckenbaugh DA, Denicoff KD (2004). Pramipexole for bipolar II depression: a placebo-controlled proof of concept study. Biol Psychiatry.

[CR397] Zetterberg H, Jakobsson J, Redsäter M, Andreasson U, Pålsson E, Ekman CJ (2014). Blood-cerebrospinal fluid barrier dysfunction in patients with bipolar disorder in relation to antipsychotic treatment. Psychiatry Res.

[CR398] Zhang P, Katz J, Michalek SM (2009). Glycogen synthase kinase-3β (GSK3β) inhibition suppresses the inflammatory response to Francisella infection and protects against tularemia in mice. Mol Immunol.

[CR399] Zheng W, Zeng Z, Bhardwaj SK, Jamali S, Srivastava LK (2013). Lithium normalizes amphetamine-induced changes in striatal FoxO1 phosphorylation and behaviors in rats. Neuroreport.

[CR400] Zhuang X, Oosting RS, Jones SR, Gainetdinov RR, Miller GW, Caron MG (2001). Hyperactivity and impaired response habituation in hyperdopaminergic mice. Proc Natl Acad Sci USA.

[CR401] Zuckerman L, Rehavi M, Nachman R, Weiner I (2003). Immune activation during pregnancy in rats leads to a postpubertal emergence of disrupted latent inhibition, dopaminergic hyperfunction, and altered limbic morphology in the offspring: a novel neurodevelopmental model of schizophrenia. Neuropsychopharmacolog.

